# Acetylsalicylic acid differentially limits the activation and expression of cell death markers in human platelets exposed to *Staphylococcus aureus* strains

**DOI:** 10.1038/s41598-017-06024-2

**Published:** 2017-07-17

**Authors:** Adrien Chabert, Pauline Damien, Paul O. Verhoeven, Florence Grattard, Philippe Berthelot, Fabrice Zeni, Laurence Panicot-Dubois, Stéphane Robert, Françoise Dignat-George, Marie-Ange Eyraud, Bruno Pozzetto, Bernard Payrastre, Fabrice Cognasse, Olivier Garraud, Hind Hamzeh-Cognasse

**Affiliations:** 1 EA3064—GIMAP, Université de Lyon, 42023 Saint-Etienne, France; 2 Laboratoire des Agents Infectieux et d’Hygiène, CHU de Saint-Etienne, 42055 Saint-Etienne, France; 3 Service de Réanimation polyvalente, CHU de Saint-Etienne, 42055 Saint-Etienne, France; 4 Université Aix Marseille, INSERM UMR-S1076, VRCM Marseille, France; 5 EFS Auvergne-Rhone-Alpes, 42023 Saint-Etienne, France; 6 Inserm/UPS UMR 1048 - I2MC, Toulouse, France; 7 Institut National de la Transfusion Sanguine, 75015 Paris, France

## Abstract

Beyond their hemostatic functions, platelets alter their inflammatory response according to the bacterial stimulus. *Staphylococcus aureus* is associated with exacerbated inflammation and thrombocytopenia, which is associated with poor prognosis during sepsis. Acetylsalicylic acid and statins prevent platelet aggregation and decrease the mortality rate during sepsis. Therefore, we assessed whether these two molecules could reduce *in vitro* platelet activation and the inflammatory response to *S. aureus*. Platelets were exposed to clinical strains of *S. aureus* in the presence or absence of acetylsalicylic acid or fluvastatin. Platelet activation, aggregation, and release of soluble sCD62P, sCD40 Ligand, RANTES and GROα were assessed. Platelet cell death was evaluated by analyzing the mitochondrial membrane potential, phosphatidylserine exposure, platelet microparticle release and caspase-3 activation. All *S. aureus* strains induced platelet activation but not aggregation and decreased the platelet count, the expression of cell death markers and the release of RANTES and GROα. Acetylsalicylic acid but not fluvastatin limited platelet activation and inflammatory factor release and restored the platelet count by protecting platelets from *Staphylococcus*-induced expression of cell death markers. This study demonstrates that acetylsalicylic acid limits *S. aureus-*induced effects on platelets by reducing cell death, revealing new strategies to reduce the platelet contribution to bacteremia-associated inflammation.

## Introduction

Platelets are chiefly known for their hemostatic role, but they have many other functions, particularly in innate immunity, host defense against infection and inflammatory processes^1–3^. Platelets act as sentinels of the vascular system due to the variety of receptors they express, such as αIIbβ3, GPIb and Toll-like Receptors (TLRs), which enable them to interact with pathogens as well as endothelial and circulating immune cells for various effector and regulatory functions^2, 4^. Several studies have demonstrated that platelets can sense pathogen-associated molecular patterns (PAMPs) through pathogen recognition receptors (PRRs)^1, 5, 6^. Moreover, platelets produce numerous biological response modifiers (BRMs) such as CD40-ligand (CD40L), a major immunoregulatory molecule during immune—including inflammatory—responses^7, 8^.

*Staphylococcus aureus* (*S. aureus*) is a pathogen that frequently causes sepsis, which can be particularly severe^9–11^. Indeed, *S. aureus* bears and releases numerous virulence factors, including enzymes and toxins^12^, which induce either the formation^13^ or the degradation of neutrophil extracellular traps (NETs) that can lead to neutrophil or macrophage apoptosis^14, 15^. Exotoxins are particularly important in the interaction between *S. aureus* and platelets. Indeed, α-hemolysin accelerates platelet-neutrophil aggregate formation^16^ or triggers B-cell lymphoma (Bcl-3) synthesis by platelets^17^. However, there are much conflicting data concerning platelet aggregation upon stimulation with *S. aureus*. Staphylothrombin, for instance, by converting fibrinogen into fibrin promotes the aggregation of platelets^18^. Similarly, Clumping Factor A, associated with specific antibodies, also induces platelet aggregation^19^. In contrast, lipotechoic acid has been shown to inhibit platelet aggregation by preventing the mobilization of intracellular calcium^20^. As very nicely described in the review by Cox *et al*.^2^, *S. aureus* can interact with platelets either directly or indirectly through plasma soluble factors, and this diversity of potentially involved mechanisms further complicates the elucidation of bacteria-induced platelet aggregation.

Sepsis is frequently accompanied by thrombocytopenia; a previous study showed that the latter follows the course of bloodstream infection and parallels adverse outcomes^21^. These findings collectively suggest that platelets have a crucial role in the pathophysiology of sepsis^22, 23^. Several mechanisms may cause this reduction in platelet count. An infectious environment can induce platelet activation and promote disseminated intravascular coagulation, which retains platelets within thrombi formed around the pathogens and thus removes them from circulation^24^. Sepsis-associated thrombocytopenia can also result from increased platelet apoptosis. Indeed, platelets have recently been reported to display features associated with apoptosis pathways such as mitochondrial membrane depolarization and caspase cascade activation^25, 26^. Indeed, increased mitochondrial membrane depolarization in platelets has been described as a biomarker of severity in sepsis^27, 28^. *S. aureus* has also been shown to induce (*in vitro*) the degradation of the survival protein Bcl-x_L_, indicating that this bacterium triggers platelet apoptosis^29^.

Molecules such as acetylsalicylic acid (aspirin, ASA) and statins have been used to reduce complications and improve survival in patients with a bloodstream infection involving *S. aureus* ^30–33^, and the antiplatelet effects of aspirin reduce severity markers in experimental *S. aureus* endocarditis^34^. However, recent studies have also shown that aspirin induces platelet apoptosis^35^ and reduces the platelet life span^36^; thus, the effects of this drug may be complex in sepsis patients^37^. Overall, it appears particularly important to counteract the occurrence of thrombocytopenia during sepsis and to identify the effects of antiplatelet molecules^38^. Elucidating the mechanisms that are collectively responsible for platelet impairment in sepsis, using ASA as a tool, can help determine previously unknown causes and may be translated into clinical solutions.

## Results

### *S. aureus* strains differentially alter the platelet count

**Figure 1:**
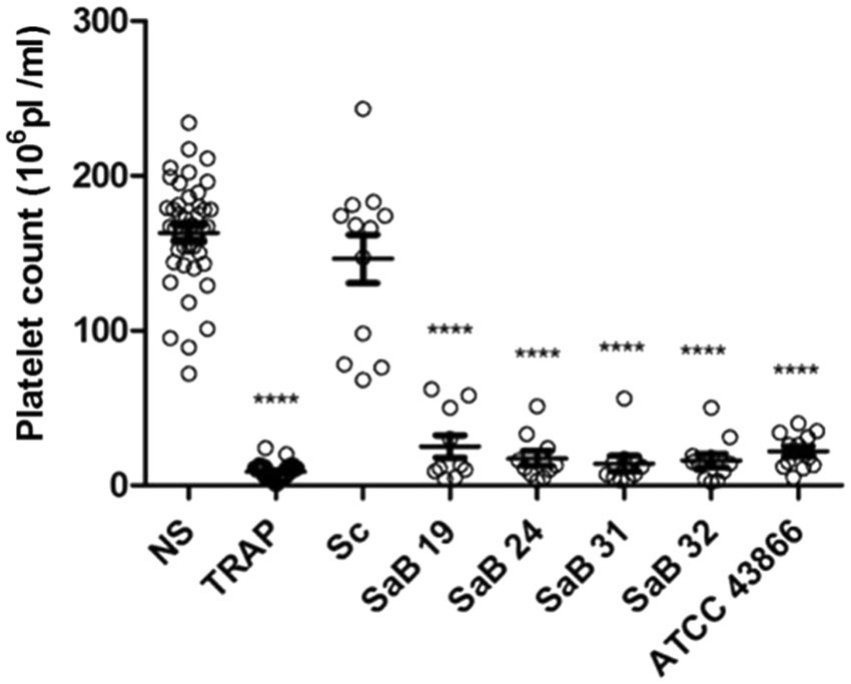
Platelet counts following exposure to *Staphylococcus* strains. After a 30-min exposure to the staphylococcal strains, TRAP (as a positive control) or Tyrode’s buffer (as a negative control, NS), platelet counts were assessed with a MS4s Hematology analyzer [Tyrode (n = 42), TRAP (n = 40) or *Staphylococcus* strains: clinical strains (SaB), *S. condimenti* (Sc) or *S. aureus* ATCC 43866 (ATCC43866)] (n = 12). The data (mean ± SEM) are expressed as the platelet count × 10^6^/ml ****p < 0.0001 (one-way ANOVA with repeated measures and the Bonferroni post hoc test; stimulated *vs*. unstimulated, NS).

The effects of several live *S. aureus* clinical strains isolated from bacteremia on normal platelets were evaluated under *ex vivo* conditions to assess the residual post-exposure counts of viable normal platelets. These strains were compared to the effects of a non-clinical, food-derived strain, *Staphylococcus condimenti*, which is poorly associated with human pathologies^39^, and a reference methicillin-resistant strain of *S. aureus*, ATCC43866. We observed that *S. condimenti* did not affect the platelet counts compared with the controls (platelets not exposed to *S. aureus* strains but to a vehicle control, Tyrode’s buffer) (146.3 ± 15.6 × 10^6^ Pl/ml *vs*. 163.2 ± 5.3 × 10^6^ Pl/ml, respectively, p > 0.05). In contrast, all clinical *S. aureus* strains isolated from bacteremia (SaB) drastically and significantly reduced platelet counts (p < 0.001) comparably to the effects of Thrombin receptor activator peptide (TRAP)-stimulation (Fig. 1). Indeed, from an initial count of 163.2 ± 5.3 × 10^6^ Pl/ml, the platelet counts decreased to a range from 14.2 ± 4.9 × 10^6^ Pl/ml to 25.2 ± 7.3 × 10^6^ Pl/ml after SaB31 and SaB19 stimulation, respectively, and reached 8.77 ± 0.8 × 10^6^ Pl/ml after stimulation with TRAP.

The reduction in platelet counts appeared to be related to an alteration in platelet morphology that prevented their automatic counting according to their size/structure by the hematological analyzer. Indeed, fluorescence microscopy experiments showed that 48% of unstimulated platelets maintained their usual size (less than 4 µm) and discoid shape (Supplemental Fig. 1a), and 31% of TRAP-stimulated platelets retained their size; the remainder spread and aggregated (Supplemental Fig. 1b), and only 21% of platelets exposed to the SaB24 bacteremia strain maintained a size less than 4 µm (Supplemental Fig. 1c). Moreover, the SaB24 strain promoted a dramatic change in morphology involving the spreading and emission of pseudopods.

### *S. aureus* strains promote platelet activation but do not lead to platelet aggregation

**Figure 2:**
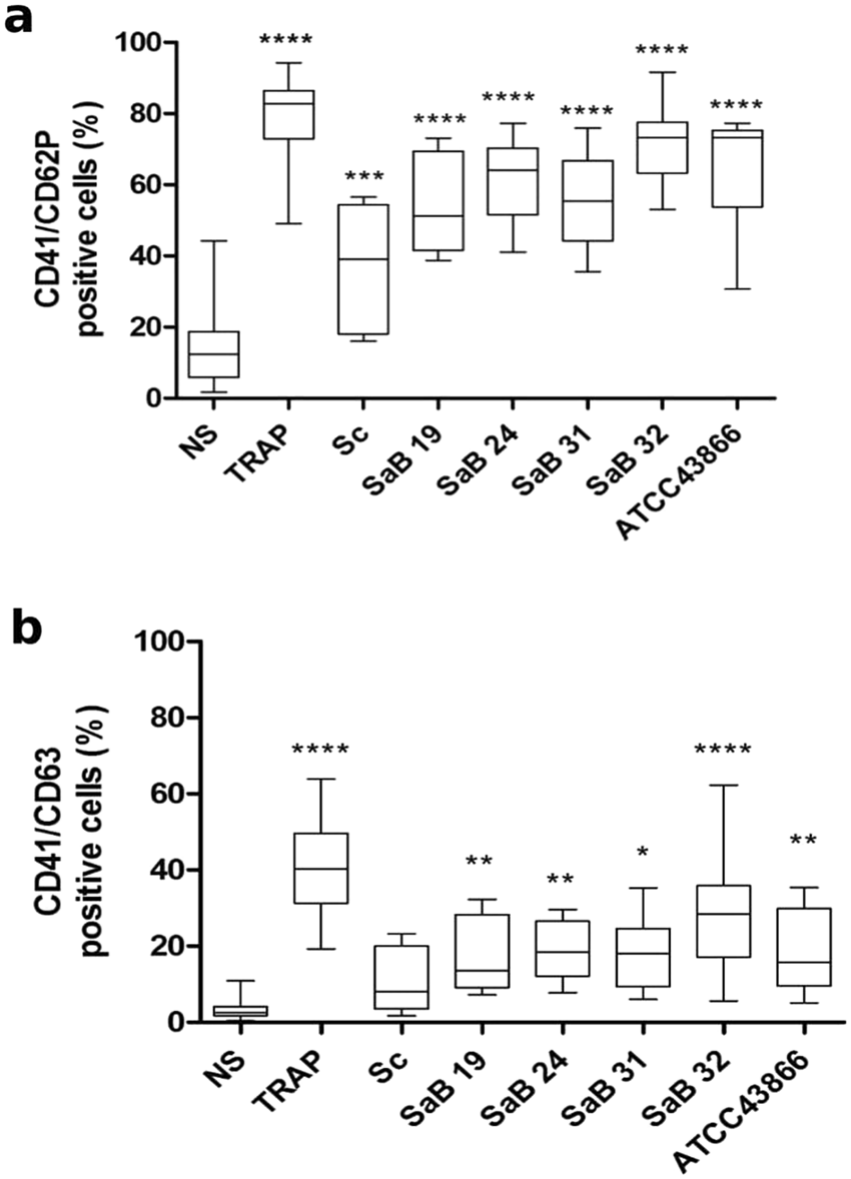
Platelet activation level after *Staphylococcus aureus* stimulation. Expression levels of the CD62P (**a**) and CD63 (**b**) activation markers on platelets were analyzed after stimulation for 30 min with staphylococcal strains [clinical strains (SaB), *S. condimenti* (Sc) or *S. aureus* ATCC 43866 (ATCC43866)], TRAP (as a positive control) or Tyrode’s buffer (as a negative control, NS). Membrane expression of CD62P and CD63 after gating on CD41 was assessed by flow cytometry, and data (mean ± SEM; n = 10 experiments) are expressed as the percentage of positive cells. *p < 0.05; **p < 0.01***p < 0.001; ****p < 0.0001 (one-way ANOVA with repeated measures and the Bonferroni post hoc test; stimulated *vs*. unstimulated, NS).

We evaluated the impact of *S. aureus* stimulation on platelets by assessing the expression of the platelet activation markers CD62P and CD63. TRAP, which was used as a positive control, induced strong and significant expression of CD62P (79.3 ± 2.3%) **(**Fig. 2a/Supplemental Fig. 2a) and CD63 (40.4 ± 2.6%) **(**Fig. 2b/Supplemental Fig. 2b). The non-pathogenic staphylococcal strain *S. condimenti* promoted the modest expression of CD62P on platelets (37.2 ± 5.4% *vs*. 15.6 ± 2.2%, p < 0.001) **(**Fig. 2a/Supplemental Fig. 2a) and no significant increase in CD63 (10.9 ± 2.6% *vs*. 3.4 ± 0.6%, p > 0.05) **(**Fig. 2b/Supplemental Fig. 2b). Clinical and reference *S. aureus* strains induced significant expression of both CD62P and CD63 (Fig. 2a and b/Supplemental Fig. 2a and b, respectively).

**Figure 3:**
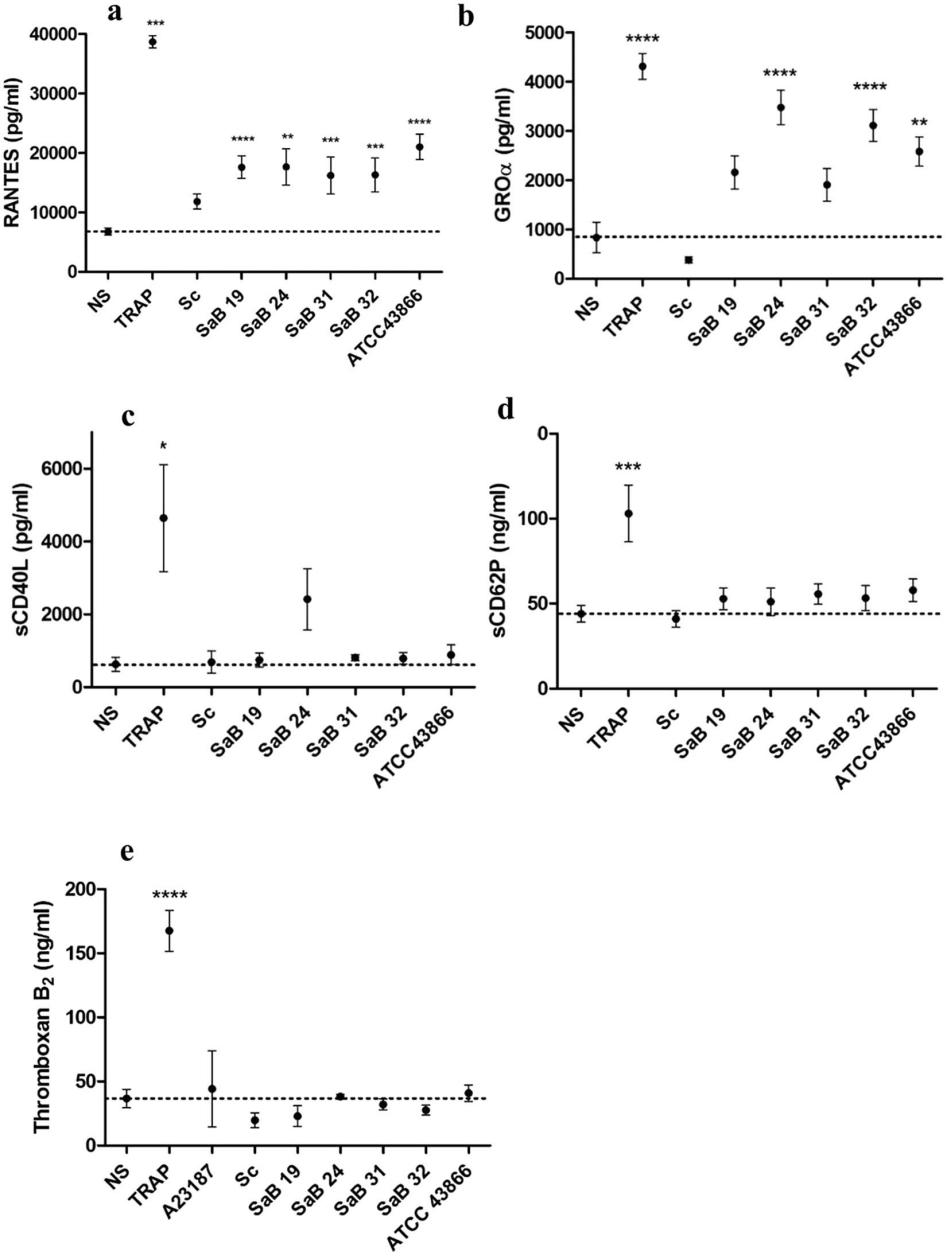
Platelet cytokine release in response to *S. aureus* stimulation. RANTES (**a**) GROα (**b**) sCD40L **(c)** sCD62P (**d**) and thromboxane B2 (**e**) release by platelets were analyzed after stimulation for 30 min with staphylococcal strains [clinical strains (SaB), *S. condimenti* (Sc) or *S. aureus* ATCC 43866 (ATCC43866)], TRAP (as a positive control) or Tyrode’s buffer (as a negative control, NS). Immunomodulatory factor levels were assessed by ELISA or Luminex® assays, and data (mean ± SEM; n = 10 experiments) are expressed as pg/ml or ng/ml (sCD62P and thromboxane B2). *p < 0.05; **p < 0.01***p < 0.001; ****p < 0.0001 (one-way ANOVA with repeated measures and the Bonferroni post hoc test; stimulated *vs*. unstimulated, NS). The dotted line represents the unstimulated value.

S*. condimenti* failed to induce any significant release of RANTES, GROα, sCD62P, thromboxane B2 or sCD40L by platelets (11816 ± 1276 pg/ml, 381 ± 62 pg/ml, 41 ± 5 ng/ml, 19.8 ± 3.5 ng/ml and 690 ± 303 pg/ml, respectively) compared with unexposed platelets (6753 ± 597 pg/ml, 836 ± 311 pg/ml, 44 ± 5 ng/ml, 36.7 ± 7.2 ng/ml and 632 ± 195 pg/ml for RANTES, GRO-α, sCD62P, thromboxane B2 and sCD40L, respectively, Fig. 3).

Every *S. aureus* strain exposed to platelets resulted in the release of significant amounts of RANTES, with levels ranging between 16222 ± 3131 pg/ml and 21023 ± 2142 pg/ml, for the SaB31 and ATCC43866 strains, respectively; this secretion is copious but was less than that induced by platelet exposure to TRAP (36688 ± 1022 pg/ml) (Fig. 3a). Platelets exposed to ATCC43866, SaB32 and SaB24 strains released significantly elevated amounts of GRO-α (2580 ± 296 pg/ml, 3112 ± 323 pg/ml and 3477 ± 348 pg/ml, respectively) (Fig. 3b). However, these levels were less than those induced by TRAP (4642 ± 1470 pg/ml vs. 836 ± 311 pg/ml in unexposed platelets, p < 0.0001, Fig. 3b). The SaB19 and SaB31 strains induced no significant release of GRO-α by platelets (Fig. 3b). All *S. aureus* strains, excluding SaB19 and SaB31, the effects of which were not statistically significant, induced a significant increase in specific cytokines, in contrast to the non-pathogenic strain *S. condimenti*.

Only SaB24 stimulation resulted in an increased production of sCD40L, although it was non-significant (4642 ± 1470 pg/ml, and 2410 ± 842 pg/ml, respectively, *vs*. 632 ± 195 pg/ml; (Fig. 3c). Neither the *S. aureus* strains nor the non-pathogenic strain *S. condimenti* altered the baseline production of sCD62p, in contrast to TRAP (103 ± 17 ng/ml compared with the unstimulated value of 44 ± 5 ng/ml, p < 0.001, Fig. 3d). The same results were observed for thromboxane B2 release (167.6 ± 15.9 ng/ml for TRAP compared with the unstimulated value of 36.7 ± 7.2 ng/ml, p < 0.001, Fig. 3e). Overall, similar to the non-pathogenic *S. condimenti* strain, all *S. aureus* strains failed to induce the release of thromboxane B2, sCD40L and sCD62P by platelets, except the SAB24 strain, which induced an increase in sCD40L release, although this increase was not significant. However, the strains succeeded in differentially mobilizing platelet stores, as demonstrated by the significant release in either RANTES or GRO-α. Thus, *S. aureus* strains altered the secretion profile of exposed platelets with different patterns rather than a unique pattern.

**Figure 4:**
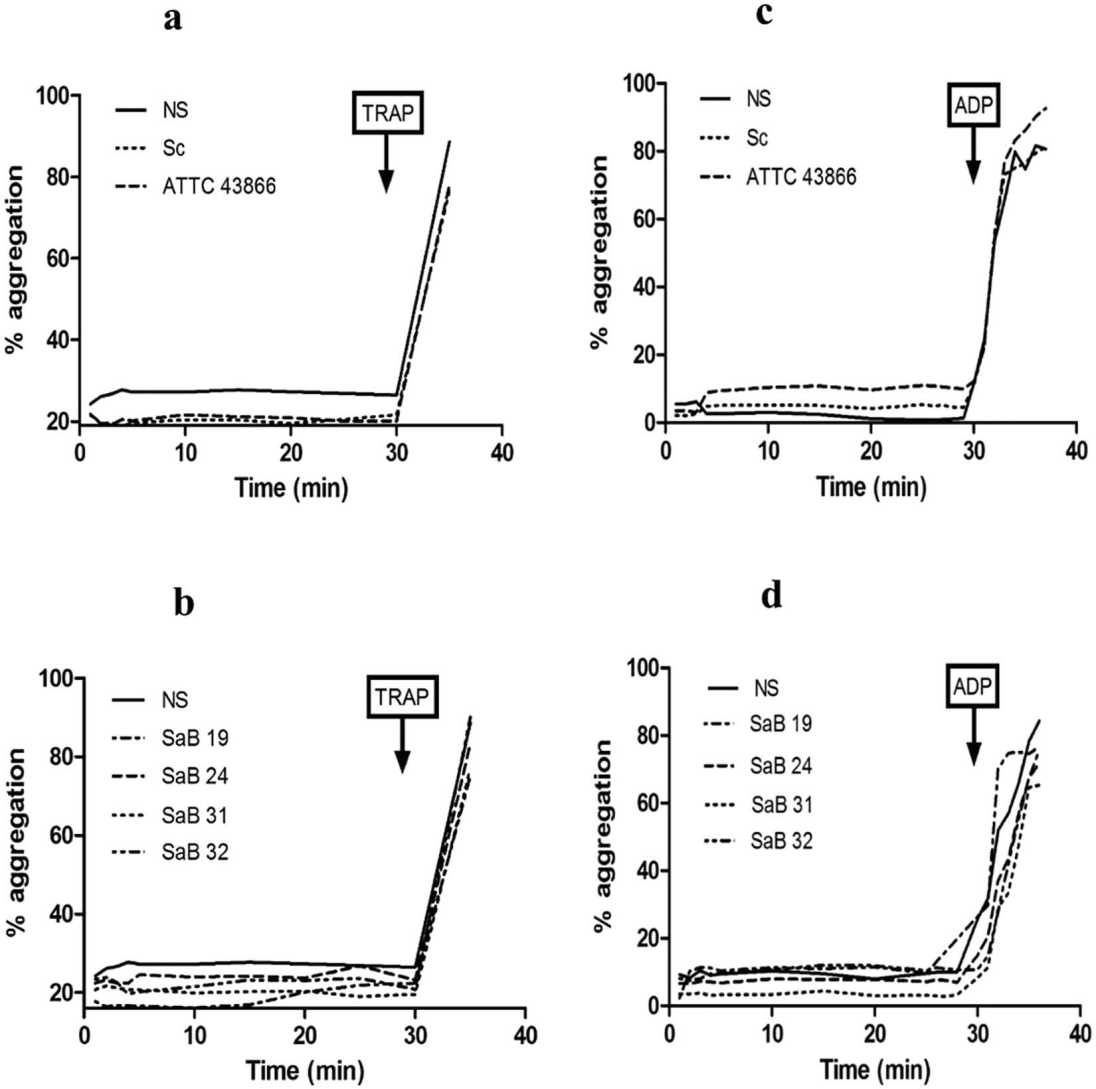
Platelet aggregation after *S. aureus* exposure. Aggregation was assessed using a thrombo-aggregometer after exposure **(a/c)** to Tyrode’s buffer (NS), *S. condimenti* (Sc) and *S. aureus* ATCC43866 (ATCC 43866) or **(b/d)** to clinical strains (SaB). TRAP **(a/b)** or ADP **(c/d)** was added as a positive control at the end of the stimulation (one representative experiment is presented; n = 5–10 experiments). The aggregation baseline was approximately 20% due to the added volume.

We next assessed whether the aggregation capacity of platelets was altered in the presence of clinical and reference *S. aureus* strains (because platelets tend to clump around bacteria to seclude them and limit infection^40^). We did not observe any platelet aggregation during a 30-min stimulation, a time frame that allowed aggregation in response to TRAP or to the weaker agonist ADP, with any staphylococcal strains (Fig. 4). Five of 35 platelet-rich plasma (PRP) samples resulted in partial aggregation **(**Supplemental Fig. 3), suggesting a modest donor dependency, and they were not included in the whole aggregation dataset.

### *S. aureus* strains promote platelet cell death

The apparent effect of clinical *S. aureus* strains on viable platelet counts, platelet activation, changes in morphology and the release of inflammatory factors suggested a robust upheaval in platelet physiology and led us to question whether exposure to bacteria *in vitro* could trigger a platelet cell death signal. To achieve this goal, we assessed membrane depolarization in platelet mitochondria using DIOC6(3) staining after exposure to various staphylococcal strains.

**Figure 5:**
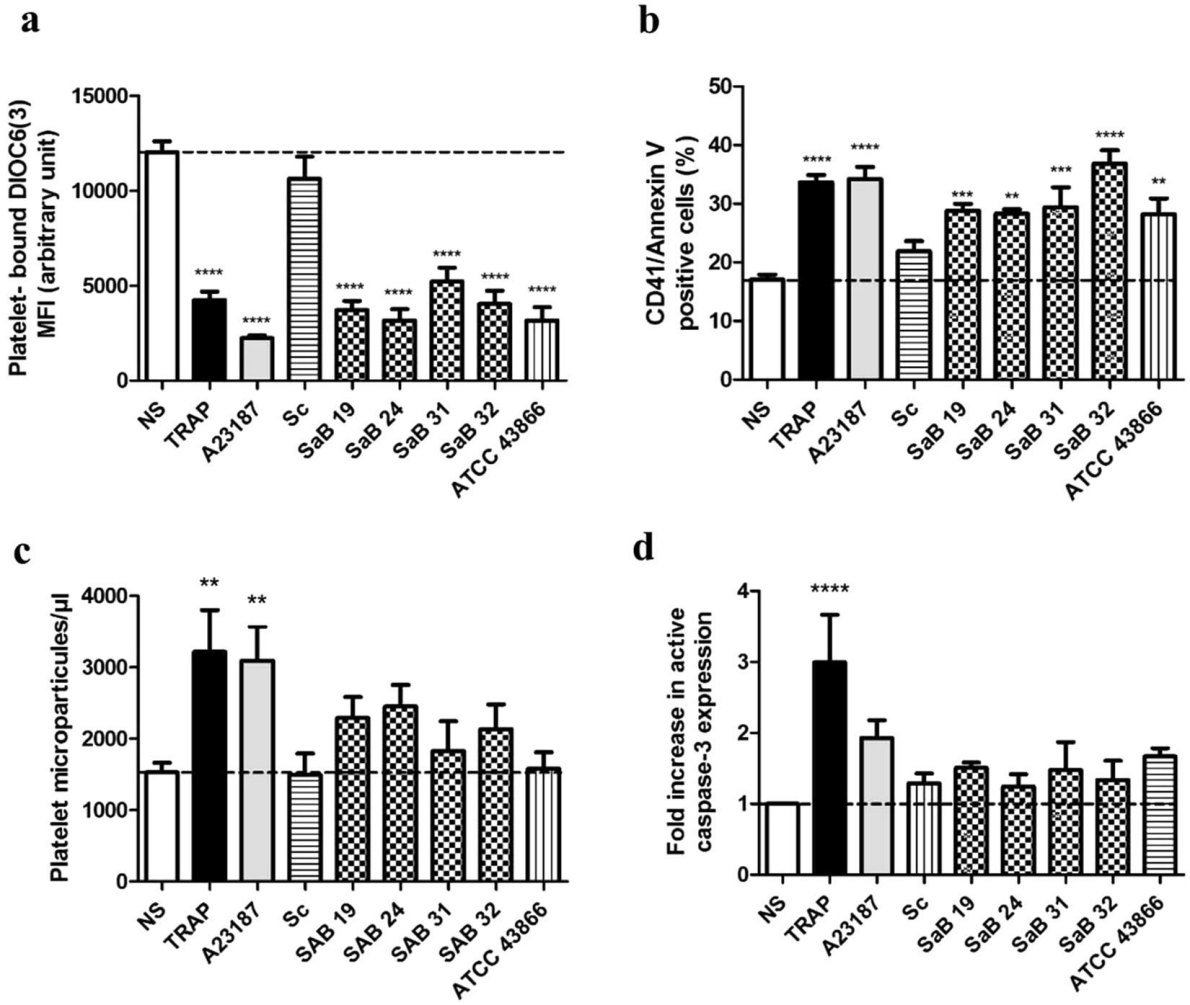
*S. aureus*-induced platelet cell death. (**a**) MFI of platelet-bound DIOC6(3) **(b)** Annexin V binding **(c)** PMP concentration or **(d)** fold increase in active caspase-3 expression were analyzed by flow cytometry after 30 min of exposure to Tyrode’s buffer (NS), TRAP, staphylococcal strains [clinical strains (SaB), *S. condimenti* (Sc) or *S. aureus* ATCC 43866 (ATCC43866)] or the A23187 apoptosis inducer as a positive control. The data (mean ± SEM; n = 8 experiments) are expressed as the AU (**a**), % expression (**b**) or fold increase compared with NS (**d**). The PMP concentration (mean ± SEM; n = 6–27 experiments) (**c)** is expressed as microparticles/µl. *p < 0.05, **p < 0.01, ****p < 0.0001 (one-way ANOVA with repeated measures and the Bonferroni post hoc test; stimulated *vs*. unstimulated, NS).

There was a significant decrease in DIOC6(3) mean fluorescence intensity (MFI) in platelets following stimulation with a TRAP agonist (4239 ± 447 arbitrary unit, AU) or the apoptosis inducer A23187 (2229 ± 151 AU, p < 0.0001) compared with the control (12019 ± 571 AU, p < 0.0001). Exposure of the platelets to all *S. aureus* strains, except *S. condimenti*, led to a significant decrease in the DIOC6(3) MFI (3704 ± 488 AU for SaB19, 3153 ± 601 AU for SaB24, 5223 ± 721 AU for SaB31, 4028 ± 700 AU for SaB32 and 3163 ± 688 AU for ATCC 43866 p < 0.0001; 10614 ± 1167 AU, for *S. condimenti*, p > 0.05) (Fig. 5a).

Next, we examined platelet phosphatidylserine (PS) exposure, which corresponds to a later stage of platelet cell death, using Annexin V binding. The TRAP agonist and apoptosis inducer A23187 promoted significant platelet expression of Annexin V (33.7 ± 1.3%, p < 0.0001; 31.1 ± 2%, p < 0.0001, respectively) compared with unstimulated platelets (16.3 ± 0.8%). Platelet Annexin V expression increased after exposure to all staphylococcal strains, except *S. condimenti* (28.4 ± 1.4% for SaB19, p < 0.005; 27.3 ± 0.7% for SaB24, p < 0.01; 31.4 ± 3.4% for SaB31, p < 0.005; 36.5 ± 2.3% for SaB32, p < 0.0001; 29.8 ± 2.7% for ATCC 43866, p < 0.01), compared with the control (16.3 ± 0.8%), indicating increased PS exposure after *S. aureus* treatment (Fig. 5b).

We also measured platelet microparticle (PMP) formation. Both TRAP and A23187 induced significant release of PMPs (3194 ± 595 PMPs/µl, p < 0.01; 3075 ± 500 PMPs/µl, p < 0.01) compared with unstimulated platelets (1576 ± 118 PMPs/µl). However, despite a non-significant increase in PMP formation in the presence of clinical strains, no difference was observed for *S. condimenti* (1405 ± 287 PMPs/µl) and ATCC 43866 (1518 ± 243 PMPs/µl), but every other strain exhibited increased PMP formation (2248 ± 283 PMPs/µl for SaB19; 2400 ± 303 PMPs/µl for SaB24; 2182 ± 326 PMPs/µl for SaB31; 2389 ± 243 PMPs/µl for SaB32), in contrast to the unstimulated platelets (1576 ± 118 PMPs/µl) (Fig. 5c). Similarly, the level of active caspase-3 increased only upon TRAP stimulation (3.0 ± 0.7-fold increase vs unstimulated control, NS, p < 0.001, Fig. 5d). Although not significant, the ATCC43866 *S. aureus* strain induced an increase in activated caspase-3 expression comparable to that triggered by the A23187 apoptosis inducer (1.7 ± 0.1-fold increase for ATCC43866 vs 1.9 ± 0.3-fold increase for A23187 stimulation).

### ASA, in contrast to fluvastatin, reduces the *S. aureus*-induced reduction in platelet counts

We next examined whether antiplatelet drugs can interfere with the effects of *S. aureus* strains on platelets. We selected two distinct antiplatelet drugs, ASA and fluvastatin, which target cyclooxygenase and 3-hydroxy-3-methylglutaryl coenzyme A reductase, respectively^38, 41^.

**Figure 6:**
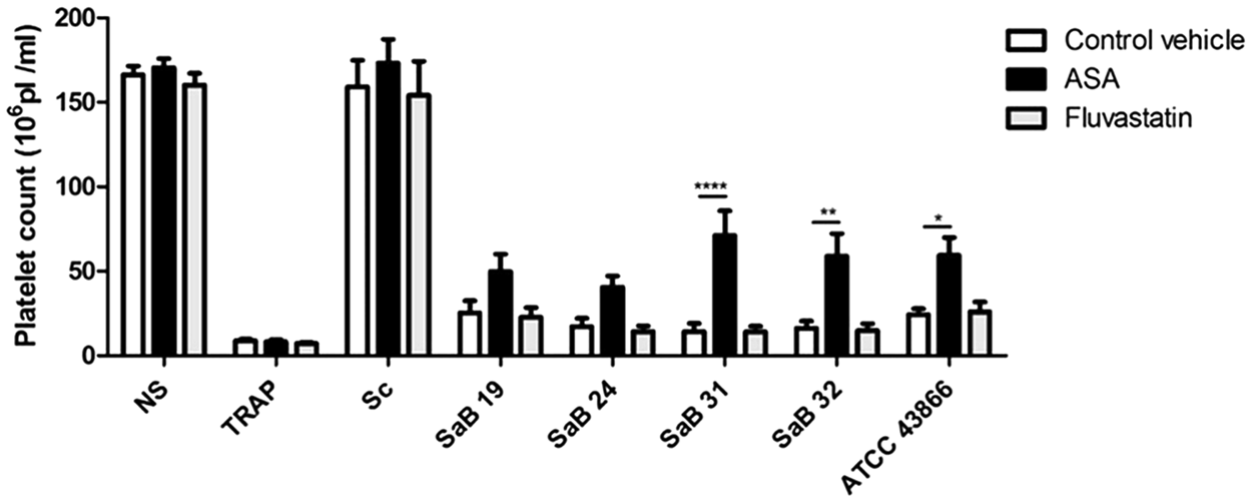
Effect of ASA and fluvastatin on the *S. aureus*-induced reduction in platelet counts. Platelet counts after a 30-min exposure to staphylococcal strains [clinical strains (SaB), *S. condimenti* (Sc) or *S. aureus* ATCC 43866 (ATCC43866)], TRAP (as a positive control) or Tyrode’s buffer (as a negative control, NS), ASA, fluvastatin or water (as a control vehicle) [control vehicle (n = 42), TRAP (n = 40) or *Staphylococcus* strains (n = 10)]. The data (mean ± SEM) are expressed as the platelet count × 10^6^/ml. *p < 0.05,**p < 0.01; ****p < 0.0001 (two-way ANOVA with repeated measures and the Bonferroni post hoc test; ASA or fluvastatin vs. control vehicle).

First, we assessed the ability of antiplatelet molecules to prevent the platelet count decrease after exposure to *S. aureus* strains. Notably, when there was no reduction in platelet counts (such as in the presence of *S. condimenti*), and neither ASA nor fluvastatin had any detectable effect. In contrast, ASA and fluvastatin did not prevent the platelet count reduction induced by platelet exposure to TRAP. Furthermore, fluvastatin did not reverse the platelet count reduction observed after the platelets were exposed to any of the clinical or reference strains of *S. aureus*. In sharp contrast, ASA partially prevented the platelet count reduction caused by platelet exposure to the SaB31, SaB32 and ATCC 43866 strains compared with the control vehicle (water) (71 ± 14.7 *vs*. 14.2 ± 4.9 × 10^6^ Pl/ml, p < 0.0001; 58.7 ± 13.6 *vs*. 16.2 ± 4.2 × 10^6^ Pl/ml, p < 0.01; 63.5 ± 9.6 *vs*. 22.2 ± 3.3 × 10^6^ Pl/ml, p < 0.01, respectively) (Fig. 6). ASA was less effective when platelets were exposed to SaB19 or SaB24 (49.7 ± 10.4 *vs*. 25.2 ± 7.3 × 10^6^ Pl/ml, and 40.5 ± 6.7 *vs*. 17.3 ± 4.7 × 10^6^ Pl/ml, p > 0.05, respectively) (Fig. 6).

### ASA limits *S. aureus*-induced platelet activation

**Figure 7:**
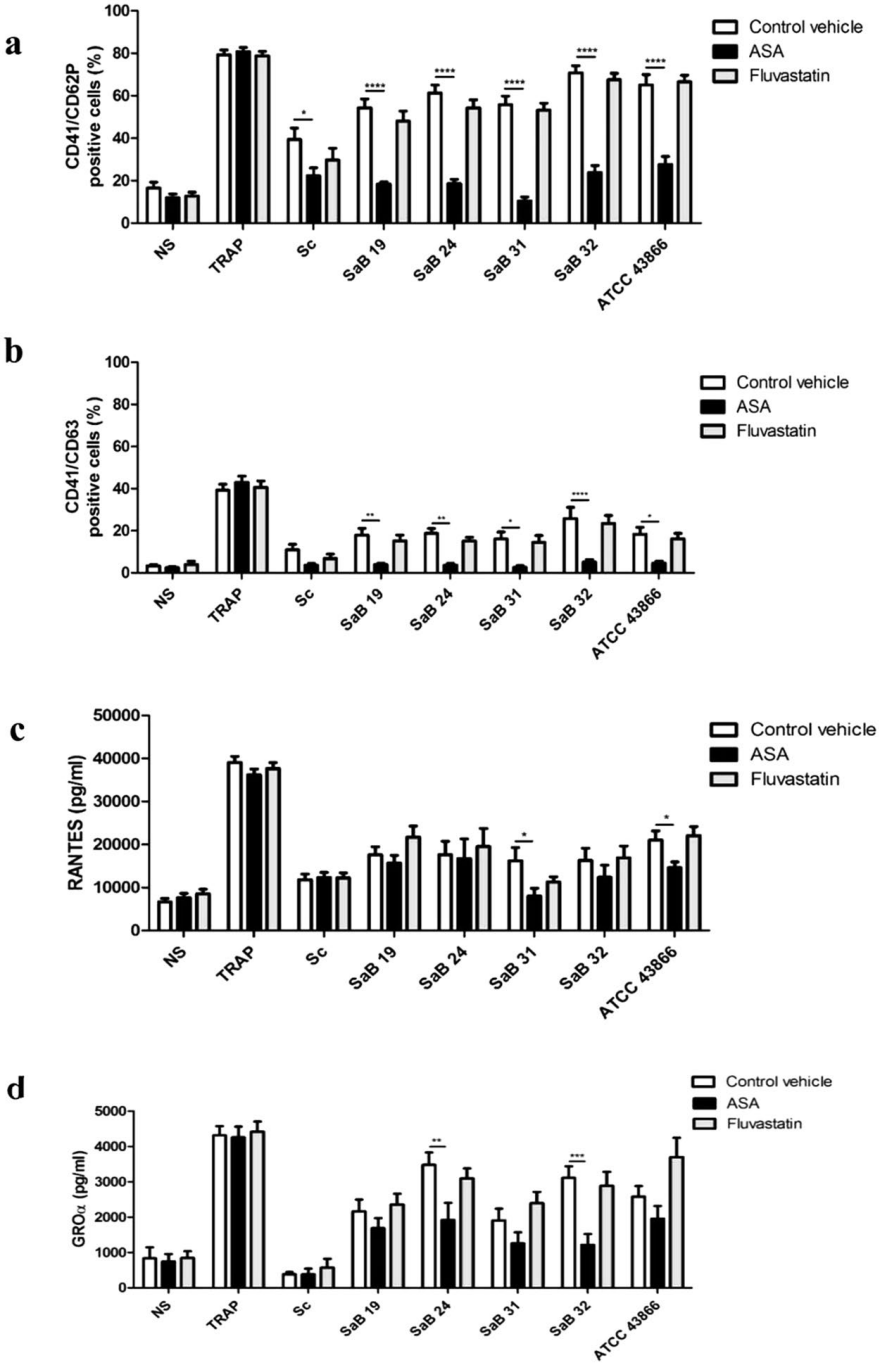
Effect of ASA and fluvastatin on *S. aureus*-induced platelet activation. Expression levels of the CD62P **(a)** and CD63 **(b)** activation markers and immunomodulatory factor release of RANTES **(c)** and GROα **(d)** by platelets were analyzed after stimulation for 30 min with staphylococcal strains [clinical strains (SaB), *S. condimenti* (Sc) or *S. aureus* ATCC 43866 (ATCC43866)] with TRAP (as a positive control) or Tyrode’s buffer (as a negative control, NS), ASA, fluvastatin or water (as a control vehicle). Membrane expression of CD62P and CD63 after gating on CD41 was assessed by flow cytometry, and data (mean ± SEM; n = 10 experiments) are expressed as the percentage of positive cells. Immunomodulatory factor levels were assessed by ELISA or Luminex® assays, and data (mean ± SEM; n = 14–30 experiments)) are expressed as pg/ml or ng/ml (sCD62P). *p < 0.05; **p < 0.01; ***p < 0.001, ****p < 0.0001 (two-way ANOVA with repeated measures and the Bonferroni post hoc test; ASA or fluvastatin *vs*. control vehicle).

Next, we examined whether ASA affects the platelet activation phenotype following platelet exposure to bacteria. We observed a significant decrease in CD62P or CD63 expression by platelets following ASA treatment after exposure to any of the *Staphylococcus* strains—the clinical ones as well as *S. condimenti* (Fig. 7a/b). Indeed, CD62P + platelet expression was reduced to 22.2 ± 3.8%, 18.4 ± 0.9%, 18.6 ± 2.0%, 10.3 ± 1.9%, 23.8 ± 3.3% and 27.6 ± 3.7% following exposure to *S. condimenti*, SaB19, SaB24, SaB31, SaB32 and ATCC 43866, respectively, in the presence of ASA, compared with 37.2 ± 5.4%, 54.3 ± 4.2%, 61.3 ± 3.7%, 55.8 ± 4.0%, 70.8 ± 3.4% and 65.0 ± 5.0% for the same strains, respectively, in the absence of ASA (p < 0.0001).

A similar phenomenon was observed for CD63 expression, which was significantly reduced in the presence of ASA, regardless of the *S. aureus* strain used. For example, the maximal CD63 expression by platelets upon *S. aureus* SaB32 exposure reached 28.2 ± 5.1% but was reduced to 5.6 ± 1.2% in the presence of ASA (p < 0.0001). Notably, fluvastatin did not reverse any of the *S. aureus*-induced activation signals assessed under these conditions (Fig. 7a/b).

Thereafter, we assessed whether ASA and fluvastatin could reduce the release of immunomodulatory factor by platelets exposed to the various *S. aureus* strains. Again, fluvastatin had no noticeable effects and did not prevent platelet secretion following exposure to clinical *S. aureus* strains (Fig. 7c–d). In contrast, ASA significantly reduced the secretion of RANTES or GROα, although for a limited number of *S. aureus* strains (Fig. 7c/d). ASA reduced the secretion of RANTES when platelets were exposed to SaB31 or ATCC43866 (8038 ± 1770 pg/ml and 14617 ± 1315 pg/ml, respectively, *vs*. 16222 ± 3101 pg/ml and 21023 ± 2142 pg/ml; p < 0.05). ASA limited the secretion of GROα by platelets when they were exposed to SAB24 and SAB32 but not to others (1920 ± 484 pg/ml and 1216 ± 305 pg/ml, respectively, *vs*. 3477 ± 348 pg/ml; p < 0.01 or 3112 ± 323 pg/ml p < 0.001) (Fig. 7d). A moderate decrease in the secretion of GROα by platelets exposed to SaB19, SaB31 and ATCC43866 was observed but remained modest and non-significant (Fig. 7d).

We next investigated whether the modest aggregation observed for the very few PRP samples (n = 5) exposed to staphylococcal stimuli was altered by either ASA or fluvastatin. Indeed, we observed that ASA but not fluvastatin efficiently protected platelets against *S. aureus*-induced aggregation (Supplemental Fig. 3). These data are limited in that the results were not universal among our platelet samples, but they indicate the potential benefit of ASA at least for platelets that aggregate in response to *S. aureus*.

### ASA efficiently protects platelets against *S. aureus*-induced cell death

**Figure 8:**
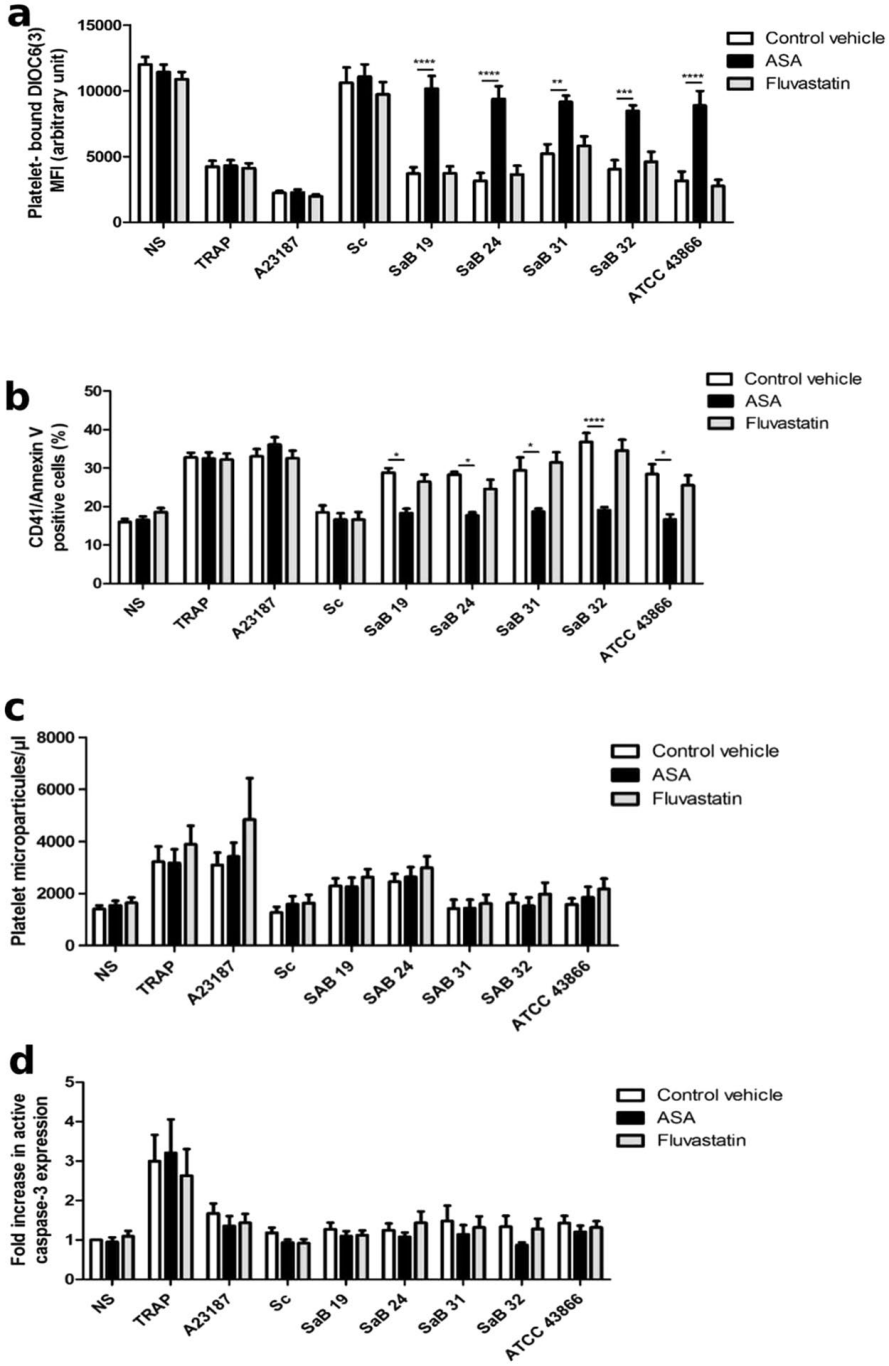
Effect of ASA and fluvastatin on *S. aureus*-induced platelet cell death. (**a**) The MFI of platelet-bound DIOC6(3) (**b**) Annexin V binding (**c**) PMP concentration or (**d**) fold increase in active caspase-3 expression were analyzed by flow cytometry after 30 min of exposure to Tyrode’s buffer (as a negative control, NS), TRAP, staphylococcal strains [clinical strains (SaB), *S. condimenti* (Sc) or *S. aureus* ATCC 43866 (ATCC43866)] or the A23187 apoptosis inducer **(**as a positive control), with ASA, fluvastatin or water (as a control vehicle). (**a**) The data (mean ± SEM; n = 8) are expressed as AU. **(b)** The data (mean ± SEM; n = 8) are expressed as the % expression (**c**) The data (mean ± SEM; n = 9–27 experiments) are expressed as microparticles/µl. The data (mean ± SEM; n = 6–15 experiments) are expressed as the fold increase in active caspase-3 expression. **p < 0.01; ***p < 0.001, ****p < 0.0001 (two-way ANOVA with repeated measures and the Bonferroni post hoc test; ASA or fluvastatin *vs*. control vehicle).

We then investigated whether ASA affects the reduction in *S. aureus*-induced platelet counts, *i.e*., induced cell death. As expected, since fluvastatin had little effect on the reduction in platelet counts in the presence of bacteria, this drug did not prevent membrane depolarization in platelet mitochondria induced by *S. aureus* (Fig. 8a). In contrast, ASA had a strong—almost complete—protective effect against *S. aureus-*induced platelet cell death. Indeed, the DIOC6(3) MFI was reduced to values between 3153 ± 601 and 5253 ± 721 AU upon bacterial exposure in the absence of treatment. However, the DIOC6(3) MFI was significantly restored to levels between 8466 ± 426 and 9390 ± 965 AU in the presence of ASA (p < 0.0001 for SaB19, SaB24 and ATCC43866 strains; p < 0.001 for SaB32 strain and p < 0.01 for SaB31 strain), which were comparable to that of unstimulated platelets (12019 ± 571 AU).

Concerning platelet PS exposure, which is usually observed during the final step of apoptosis and during necrosis^42^, ASA reduced platelet Annexin V expression induced by all *S. aureus* strains but showed no effect on platelets exposed to *S. condimenti* since this strain did not induce significant PS exposure. Indeed, the percentage of cells with CD41/Annexin V decreased from values between 27.4 ± 0.7% and 36.6 ± 2.3% after staphylococcal exposure alone to 16.7 ± 1.4% and 19.1 ± 0.8% in the presence of ASA (p < 0.0001 for SaB32, p < 0.05 for SaB19, SaB24, SaB31 and ATCC43866 strains, Fig. 8b). These levels were comparable to those of unstimulated platelets (16.57 ± 0.9%), indicating a strong protective effect of ASA on platelet cell death induced by *S. aureus* strains.

Finally, no difference was observed for each *Staphylococcus* strain in the presence of ASA with respect to the release of PMPs (Fig. 8c) or the expression of active caspase-3 (Fig. 8d). The presence of fluvastatin had no effect either on the formation of PMPs or on the expression of active caspase-3.

## Discussion

We found a pathogenic effect of clinical and reference strains of *S. aureus* on platelets from healthy donors, while *S. condimenti*, a food-derived strain associated only once with bloodstream infection^39^, had a moderate effect. Indeed, *S. aureus* is predominantly reported during bacteremia and causes a variety of infections that frequently result in death^9, 10^. We observed that freshly obtained platelets exposed to clinical and reference *S. aureus* strains for 30 min showed an altered morphology and expressed activation markers, such as CD62P and CD63, with a strong effect on CD62P, regardless of the strains used. Genotyping of the strains revealed that while some genes associated with virulence were present in all strains, for instance *Clf*A/B, *Fnb*A/B, *Luk*S/F, *Hla*, others, such as *Cna Ent*A or *Sas*G, were expressed only by some strains (Supplemental Table 1). However, no strain appeared to have a more pronounced effect than another on the platelet response. Thus, the use of isogenic mutants to examine the participation of each virulence factor in the induction of the platelet response would be helpful. In addition, none of the tested strains induced platelet aggregation, except for 5 donors. Indeed, strain SAB24 induced aggregation in 2 donors, as did strain SAB32, whereas strain ATCC43866 induced the aggregation of only one donor. Therefore, the host specificity of the response should also be taken into account.

Concerning immunomodulatory factors, every strain of *S. aureus* but not *S. condimenti* induced significant release of RANTES and of GROα for some strains. Thus, platelets exposed to *S. aureus* strains could actively contribute to the elevated plasma levels of RANTES and GROα that have been described during the course of *S. aureus* bloodstream infection and sepsis^43, 44^. GROα levels have also been shown to correlate with several clinical scores (Pediatric Risk of Mortality, Sepsis-related Organ Failure Assessment and Disseminated Intravascular Coagulation scores)^45^. Regarding RANTES, murine models of lipopolysaccharide-induced acute lung injury^46^ and of polymicrobial sepsis^47^ revealed the involvement of platelet-derived RANTES in lung damage, underscoring the contribution of this chemokine and the potential implication of platelets in sepsis-associated complications. Interestingly, we observed in our study that in the presence of ASA, the expression of activation makers on platelets was restored to unstimulated levels, and the release of RANTES and GROα was reduced.

Given that we used a multiplicity of infection (MOI) of 0.1 and that the bacterial suspension was washed to remove soluble bacterial products, it was likely that platelet activation was propagated by thromboxane B2 released by platelets since ASA could reduce this activation. However, we did not observe any release of this factor in the presence of *S. aureus* strains. This finding suggests that under the present conditions, ASA either might reduce prostaglandin E2 rather than thromboxane B2, or exert Cox-independent effects, as observed for NF-κB, although described for high doses of ASA^48^.

In addition to their well described PS-related procoagulant activity^49^, platelet microparticles have been shown to be predominantly found in the circulation during sepsis^50^, to have an inflammatory role in rheumatoid arthritis^51^ and also to have immunosuppressive properties^52, 53^. Therefore, their contribution to the inflammatory/immunosuppressive component of sepsis should not be underestimated. Although not significant under our conditions, probably because of a requirement for a longer contact duration, the increased release of platelet microparticles following *S. aureus* exposure could synergize with the release of RANTES and GROα to promote an immunomodulatory environment, contributing to sepsis pathophysiology.

These data, on the one hand, confirm that platelet secretion triggered by exposure to infectious pathogens is not “all or none,” as diverse *S. aureus* strains elicited different secretion profiles, and on the other hand, show that platelet inhibitors such as ASA also have differential effects on these secretion patterns. These results support previous studies showing that human platelets have the capacity to differentially detect external signals and adapt their inflammatory responses^54–56^. In addition to their hemostatic role, platelets have an important inflammatory function. In fact, platelets are sentinels in the circulation due to the number of immunoreceptors expressed on their surface (FcγRII, Toll-like receptors, complement receptor or αIIbβ3), which detect pathogens and participate in inflammation. Moreover, platelets interact with macrophages and neutrophils, leading to pathogen phagocytosis and NET formation^4, 57^. Although many studies have investigated the effects of bacteria on platelet hemostatic responses^58–60^, few studies have focused on their immunoregulatory role^61, 62^, although this could be a potential target to reduce bacterial sepsis^38^.

Previous studies have reported platelet apoptosis in response to *Escherichia coli* or peptidoglycan, a component of *S. aureus* cell walls^29, 63^. In this study, we showed that in contrast to the food-derived *S. condimenti* strain, exposure to clinical or reference strains of *S. aureus* promoted PS exposure and mitochondrial membrane depolarization, which are features of both apoptosis and necrosis in platelets. However, the slight increase in both PMP release and active caspase-3 expression, although not significant, suggests the initiation of an apoptotic process that might require a longer exposure to *S. aureus* strains but does not exclude the occurrence of a necrotic process. Indeed, it has previously been elegantly shown that mitochondrial membrane depolarization and PS exposure were induced either by an apoptosis inducer or by the thrombin/collagen combination that induces necrotic platelets^42^. Thus, the use of a cell death marker, such as GSAO, associated with the expression of CD62P would help discriminate between apoptosis and necrosis under our conditions.

Conversely, we did not observe platelet aggregation during staphylococcal strain exposure, indicating that platelets have the capacity to distinguish inflammatory from aggregation responses depending on the bacterial types^59, 64^ or concentration^65^. However, there remains some controversy over the induction of platelet aggregation by *S. aureus* or its compounds. Several very nice studies report that *S. aureus* promotes platelet aggregation through Clumping Factor A^60^, which binds fibrinogen and IgG and activates the platelet GPIIb/IIIa and FCγRIIa receptors through the thromboxane A2 and ADP pathways^66^. However, platelets also harbor innate immunity receptors, such as TLR2 and TLR4, which, when triggered, may^66, 67^ or may not^20, 68^ lead to platelet aggregation. These phenomena raise questions regarding the platelet response according to the receptor engaged by bacteria and, more likely, the synthesis of the signalling pathways within platelets when several receptors are simultaneously engaged due to the multiplicity of potentially involved ligand/receptor pairs. Moreover, it is also important to consider the variability in virulence factor expression by bacterial strains because some, such as staphylothrombin, may promote aggregation^18^, while others, such as alpha-hemolysin, may inhibit it, as elegantly demonstrated by Powers *et al*.^69^. Finally, parameters of the host might also intervene, as observed in our study showing that under the same stimulation conditions, platelets from 5 of 35 donors aggregated in response to 3 of our strains.

We also evaluated the ability of molecules with marked antiplatelet activity, such as ASA and fluvastatin, to reduce platelet activation and secretion of inflammatory factors and potentially also limit platelet cell death^30–33, 70^ and PMP release^71^. Regarding statins, we observed that fluvastatin had no inhibitory effect on platelets after any *in vitro S. aureus* strain exposure, whereas recent studies have shown an inhibition of platelet hemostatic function with fluvastatin via downregulation of thromboxane A_2_ platelet synthesis^72–74^. Statins such as fluvastatin, but particularly simvastatin, have shown a protective effect against abdominal sepsis predominantly by improving endothelial function and decreasing pulmonary inflammation^75^. Additional studies are needed to understand the effects of statins on platelet function during sepsis. According to Merx *et al*., enhanced nitric oxide and cyclic GMP formation could play a role in the antiplatelet activity of simvastatin after sepsis induction^70^.

In contrast, in the present study, the administration of ASA (500 µM) was shown to modify platelet functions with a protective effect on the decrease in platelet count, a significant decrease in the activation phenotype and complete protection against platelet-induced cell death after exposure to clinical or reference *S. aureus* strains. Platelet cell death, whose mechanism could be related to apoptosis or necrosis^42^, would thus be implicated in the decreased platelet counts that occur during *S. aureus* bacteremia. Conversely, it has been shown that ASA (used at 5 mM, 10 times more concentrated than in our study) induced platelet apoptosis via the upregulation of pro-apoptotic proteins or mitochondrial pathways^35, 36^.

Platelet cell death induced by *S. aureus* strains can promote deleterious responses during sepsis via different mechanisms: (1) thrombocytopenia, which represents an important complication of sepsis; (2) immunomodulatory factors present in platelets or in microparticles, which are secreted and participate in the inflammatory phase of sepsis; and (3) the reduction in ‘vital NETosis’, in which platelet-induced NETs help capture and clear bacteria^76^. However, the detrimental role of NETs should not be underestimated because NETs have been shown to enhance thrombosis^77^, although new evidence tends to distinguish the effects of intact NETs from those of DNA and histones in the induction of coagulation^78^.

Due to their dual hemostatic and inflammatory roles, platelets could be targeted by ASA to limit the harmful effects of *S. aureus* on platelets and improve clinical outcomes in *S. aureus*-induced bacteremia^79, 80^. However, in the lipopolysaccharide-induced sepsis model in mice, ASA pretreatment reduced the survival rate by inhibiting the protective effect of platelets on macrophage-dependent inflammation^81^. Thus, more studies with more complex models are needed to better understand the inflammatory role of platelets during sepsis. Drugs such as ASA that have direct and indirect effects on platelets may be promising treatments to limit the severity of clinical sepsis; however, further studies are needed.

## Methods

### Ethical considerations

In accordance with the French Public Health Code (article L. 1223-3) and the procedures of the French Blood Bank ethical board (Etablissement Français du Sang – EFS), volunteer blood donors signed the donation form indicating that they did not preclude the use of their donation for non-therapeutic purposes. Thus, in accordance with the Declaration of Helsinki, informed and written consent has been obtained from all the healthy donors who participated in this study for the sampling of blood for scientific purposes. Analyses have been performed anonymously, in accordance with the relevant guidelines and regulations of the Lyon University (UJM, Faculty of Medicine, Saint-Etienne, France).

### Preparation of platelets and Staphylococcus strains

PRP was prepared as previously described^82^. Briefly, peripheral blood was collected from healthy donors in endotoxin-free tubes with 3.2% sodium citrate (Becton Dickinson, San Jose, CA, USA) and centrifuged at 150 × *g* for 10 min at 22 °C. There was no detectable contamination of platelets by residual mononuclear cells as assessed by flow cytometry (CD3-T cells, CD19-B cells, or CD14-monocytes) (Supplemental Fig. 4). Platelets were counted in freshly prepared PRP with a MS4s Hematology analyzer (Melet Schloesing, Osny, France), and cell suspensions were adjusted to 3 × 10^8^ Pl/ml with Tyrode’s buffer (Sigma-Aldrich, Saint Quentin-Fallavier, France) containing 0.04 U/ml of apyrase (Sigma-Aldrich, Saint Quentin-Fallavier, France) and 0.3 µg/ml of PGI_2_ (Sigma-Aldrich, Saint Quentin-Fallavier, France).

The tested staphylococcal strains were the *S. aureus* ATCC43866 reference strain, 4 *S. aureus* clinical strains isolated from bacteremia patients (SaB) 19, 24, 31, 32 and the non-pathogenic food-derived *S. condimenti* (SC) strain. For each experiment, the bacterial strains were plated, from frozen cryobead stocks, on blood agar plates and incubated overnight at 37 °C. Then, after checking for the absence of contaminating colonies and variants, a Mueller-Hinton broth was seeded with a small number of colonies and cultured under agitation for 24 h at 37 °C. A Mueller-Hinton broth was then seeded with 2 ml of the 24 h-culture and incubated at 37 °C for 4 h to reach the exponential phase. Bacterial cells were washed, resuspended in Tyrode’s Buffer and adjusted to 1.2 × 10^8^ CFU/ml.

### Platelet exposure to Staphylococcus strains

Staphylococcal strains (50 µl, corresponding to 6 × 10^6^ CFU) or Tyrode’s Buffer (50 µl, as a non-stimulated (NS) control) were added to freshly prepared platelets (200 µl, corresponding to 6 × 10^7^ platelets) in a 96-well uncoated polystyrene culture microplate to obtain a multiplicity of infection (MOI) of 0.1 (*i.e*., 1 bacteria for 10 platelets). Next, 50 µl of either ASA (500 µM final concentration, Sigma-Aldrich, Saint Quentin-Fallavier, France), fluvastatin (10 µM final concentration, Sigma-Aldrich, Saint Quentin-Fallavier, France) or control vehicle (water, the resolubilization liquid for both ASA and fluvastatin) were added to the suspension. The concentrations of ASA and fluvastatin used *in vitro* to antagonize the effects of staphylococcal stimulation were determined by stimulating the platelets with the ATCC43866 reference strain in the presence of increasing concentrations of antiplatelet molecules, ranging from 0 to 5000 µM for ASA and 100 µM for fluvastatin, based on previous studies^72, 83^. The lowest concentration of antiplatelet molecule that inhibited RANTES release by platelets was chosen for further experiments (i.e., 500 µM final concentration, Supplemental Fig. 5). However, 10 µM fluvastatin failed to inhibit RANTES release by platelets and was therefore used as a control for further experiments. The culture plate containing the platelet and bacterial mix was then gently centrifuged for 5 min at 300 × *g* to facilitate and standardize contact between platelets and bacteria. The plate was then incubated, without stirring, for 30 min at room temperature. As positive controls for either activation or platelet cell death, platelets were also stimulated for 30 min at room temperature with 50 µg/ml thrombin receptor activator peptide (TRAP-SFLLRN, Sigma-Aldrich, Saint Quentin-Fallavier, France) or with the apoptosis inducer A23187 (Merck Millipore, Guyancourt, France), respectively.

After stimulation, platelets were resuspended by gentle pipetting, and an aliquot of the suspension was collected for viable normal platelet count and flow cytometry analysis. The plate was then re-centrifuged at 300 × *g* for 10 min to harvest the platelets, and the supernatants were collected and kept frozen at −80 °C until analysis.

### Assessment of platelet mitochondrial membrane potential (ΔΨm)

To determine whether platelet exposure to staphylococcal strains induced platelet cell death, the mitochondrial membrane potential (ΔΨm) was assessed in platelets. After bacterial stimulation, 40 nM DiOC6(3), a cell-penetrable green fluorescent cationic dye (Invitrogen, Saint Aubin, France), was added to the cell suspension for 15 min at room temperature. The samples were then diluted in phosphate-buffered saline (PBS) prior to flow cytometry analysis on a CANTO II flow cytometer with BD FACSDiva™ software (BD Biosciences, Le pont de Claix, France). Depolarization of the mitochondria was defined as a decrease in the mean fluorescence intensity of platelet-bound DIOC6(3), as observed in the FL1 channel.

### Assessment of platelet phosphatidylserine externalization

Phosphatidylserine exposed on the membrane of apoptotic or necrotic cells was quantified by flow cytometry. In brief, after stimulation, platelets were resuspended in Annexin Binding Buffer (MabTag, Oldenburg, Germany) for immunostaining. PS exposure was determined after gating on CD41-positive cells (CD41 is a specific marker for platelets). Allophycocyanin (APC)-conjugated anti-human Annexin V (clone), fluorescein isothiocyanate (FITC) anti-human CD41 (clone HIP-8) and fluorochrome-conjugated mouse IgG isotypic controls were purchased from BD Biosciences (Le pont de Claix, France). All analyses were performed with a CANTO II flow cytometer and analyzed with BD FACSDiva™ software (BD Biosciences).

### Platelet microparticle preparation and flow cytometry analysis

After stimulation, PRP was centrifuged at 1,100 × g for 15 min to obtain platelet-poor plasma (PPP) containing PMPs. Then, PPP was centrifuged at 7,000 × g for 3 min. Supernatants were collected and kept frozen at −80 °C until flow cytometry analysis. PMPs were measured using Gallios flow cytometry as previously described by Robert *et al*.^84^. Briefly, PMPs were defined as CD41 and Annexin V positive, gating with Megamix beads (Biocytex, Marseille, France). PMPs were labeled following a 30-min incubation of PPP with APC-conjugated anti-human (MabTag, Oldenburg, Germany) and FITC anti-human CD41 (BD Biosciences) or fluorochrome-conjugated mouse IgG isotypic controls. During immunostaining, Annexin binding buffer was added to improve the binding of Annexin–V to PS. PMPs were fixed by addition of paraformaldehyde (4%); Cytocount beads (Dako/Agilent, Les Ulis, France) were used to determine the concentration. All analyses were performed using Kaluza® flow analysis software.

### Assessment of caspase-3 activation

Stimulated platelets were resuspended in BD Cytofix/Cytoperm™ solution (BD Biosciences, Le pont de Claix – France) and incubated for 20 min on ice according to the manufacturer’s instructions. Platelets were then centrifuged 300 × *g* for 5 min, and the pellet was washed twice with BD Perm/Wash™ buffer. Platelets were resuspended in Perm/Wash™ buffer, stained with 20 µl of FITC rabbit anti-active Caspase-3 antibody (BD Biosciences) for 30 min at room temperature, washed once and analyzed with a CANTO II flow cytometer and BD FACSDiva™ software (BD Biosciences).

### Assessment of cytokine release by platelets

The release of sCD62P, RANTES, GROα and sCD40L by platelets was measured in duplicate in the supernatants of unstimulated (negative control) or TRAP-stimulated (positive control) platelets and in the supernatants of platelets exposed to staphylococcal strains in the presence or absence of ASA (500 µM) or fluvastatin (10 µM). RANTES and Thromboxane B2 were quantified by ELISA (R&D Systems Europe Ltd., Lille, France). The absorbance at 450 nm was measured on an ELISA reader (Magellan software, Sunrise™, Tecan Group Ltd., Lyon, France). Soluble CD40L, sCD62P and GROα were quantified using a multiplexed Luminex® system (Merck Millipore, Guyancourt, France) according to the manufacturer’s instructions. The results were read using a Bioplex 200 system (Bioplex Manager™ software, Bio-Rad, Marnes-la-Coquette, France).

### Assessment of platelet aggregation

Platelet aggregation was assessed throughout the platelet stimulation (30 min) with the staphylococcal strains described above using a Thrombo-aggregometer TA 4 V with constant stirring and was analyzed with Thrombosoft 1.6 software (SD Innovation, Frouard, France). At the end of the platelet stimulation, TRAP (50 µg/ml) or ADP (10 µM) was added to the PRP suspension to confirm that the platelets were still responsive to stimulation. Calibration of the thrombo-aggregometer utilized PRP (0% light transmission, 0% aggregation) and PPP (100% light transmission, 100% aggregation), which was obtained by centrifuging the unstimulated PRP at 3000 × *g* for 20 min. The data for the PRP samples that revealed an aggregation, even partial, was not included in the whole aggregation dataset and were presented individually, as shown in Supplemental Fig. 3.

### Analysis of platelet activation markers

Platelet membrane expression of the CD62P and CD63 activation markers after stimulation was assessed using immunostaining of PRP after gating on CD41-positive cells (CD41 is a specific marker for platelets). APC-conjugated anti-human CD41 (clone HIP-8), FITC anti-human CD62P (clone AK-4), phycoerythrin (PE) CD63 (clone HSC-6) and fluorochrome-conjugated mouse IgG isotypic controls were purchased from BD Biosciences (Le pont de Claix, France). All analyses were performed with a CANTO II flow cytometer and analyzed with BD FACSDiva™ software (BD Biosciences).

### Platelets and S. aureus fluorescence microscopy

The staphylococcal strain SAB24 expressing eGFP, obtained by transforming the SAB24 strain with the pbSU101 plasmid (a kind gift from Prof. Spellerberg, University of Ulm, Germany) as previously described^85, 86^, TRAP (as a positive control) or Tyrode’s Buffer (as a non-stimulated (NS) control) were added to freshly prepared platelets as described in the “Platelet exposure to Staphylococcus strains” paragraph and placed in a μClear® polystyrene microplate with a bottom thickness of 190 μm +/− 20 μm (Greiner Bio-One, Courtaboeuf, France). The culture plate containing the platelet and bacterial mix was then gently centrifuged for 5 min at 300 × *g* to facilitate and standardize contact between platelets and bacteria and was incubated for 30 min at room temperature. The platelets were then fixed with 4% paraformaldehyde, washed twice with PBS and stained with an anti-human CD41 Dylight 550 antibody (Leinco Technologies®/Interchim, Montluçon, France; 10 µg/ml). Finally, the platelets were washed once with PBS and observed with an IX81 fluorescence inverted microscope (Olympus, Germany) using Cell^P software (Olympus, Germany). The total number of platelets and the number of platelets with a normal size (less than 4 μm) was determined in each field, and the percentage of normal-sized platelets was calculated.

### Measurement of endotoxin levels in PRP

The absence of any contaminating bacterial endotoxin in PRP was confirmed by inoculation of 1 ml of PRP into conventional blood culture medium, incubation in a BD Bactec™ automated system (Becton Dickinson) and analysis using the QCL-1000 Limulus amoebocyte lysate assay® (Lonza/Ozyme, Montigny-Le-Bretonneux, France).

### Statistical analysis

One or two-way ANOVA with repeated measures and a Bonferroni post hoc test were used for inter-experimental comparisons. All values are reported as the mean ± standard error of the mean (SEM). A p-value of <0.05 was considered statistically significant. Dixon tests were used to identify abnormal values. Statistical analyses and graphs were performed with GraphPad Prism 5 (La Jolla, USA) and FlowJo V10 (Ashland, USA) software.

## Electronic supplementary material

Supplementary Information
