# Supplementary material for: Acetylsalicylic acid differentially limits the activation and expression of cell death markers in human platelets exposed to Staphylococcus aureus strains
Source: Sci Rep. 2017 Jul 17;7:5610. doi: 10.1038/s41598-017-06024-2 (PMC5514152; doi:10.1038/s41598-017-06024-2)
Supplement: Supplementary file 1 — Supplementary Information [file 41598_2017_6024_MOESM1_ESM.pdf]

Acetylsalicylic acid differentially limits the activation and expression of cell death markers in human platelets exposed to *Staphylococcus aureus* strains

Adrien Chabert[1], Pauline Damien[1], Paul O. Verhoeven[1,2], Florence Grattard[1,2], Philippe Berthelot[1,2], Fabrice Zeni[1,3], Laurence Panicot-Dubois[4], Stéphane Robert[4], Françoise Dignat-George[4], Marie-Ange Eyraud[5], Bruno Pozzetto[1,2], Bernard Payrastre[6], Fabrice Cognasse[1,5], Olivier Garraud[1,7] and Hind Hamzeh-Cognasse[1*]

[1] *EA3064—GIMAP, Université de Lyon, 42023 Saint-Etienne, France*

[2] *Laboratoire des Agents Infectieux et d'Hygiène, CHU de Saint-Etienne, 42055 Saint-Etienne, France*

[3] *Service Réanimation polyvalente, 42055 CHU de St Etienne, France*

[4] *Université Aix Marseille, INSERM UMR-S1076, VRCM, Marseille, France*

[5] *EFS Rhone-Alpes-Auvergne, 42023 Saint-Etienne, France*

[6] *Inserm/UPS UMR 1048 - I2MC, Toulouse, France*

[7] *Institut National de la Transfusion Sanguine, 75015, Paris, France*

Correspondence to: Hind Hamzeh-Cognasse, EA3064—GIMAP, Université de Lyon, 42023 Saint-Etienne, France; Hind.Hamzeh@univ-st-etienne.fr

Supplemental data

Supplemental Figure 1

a

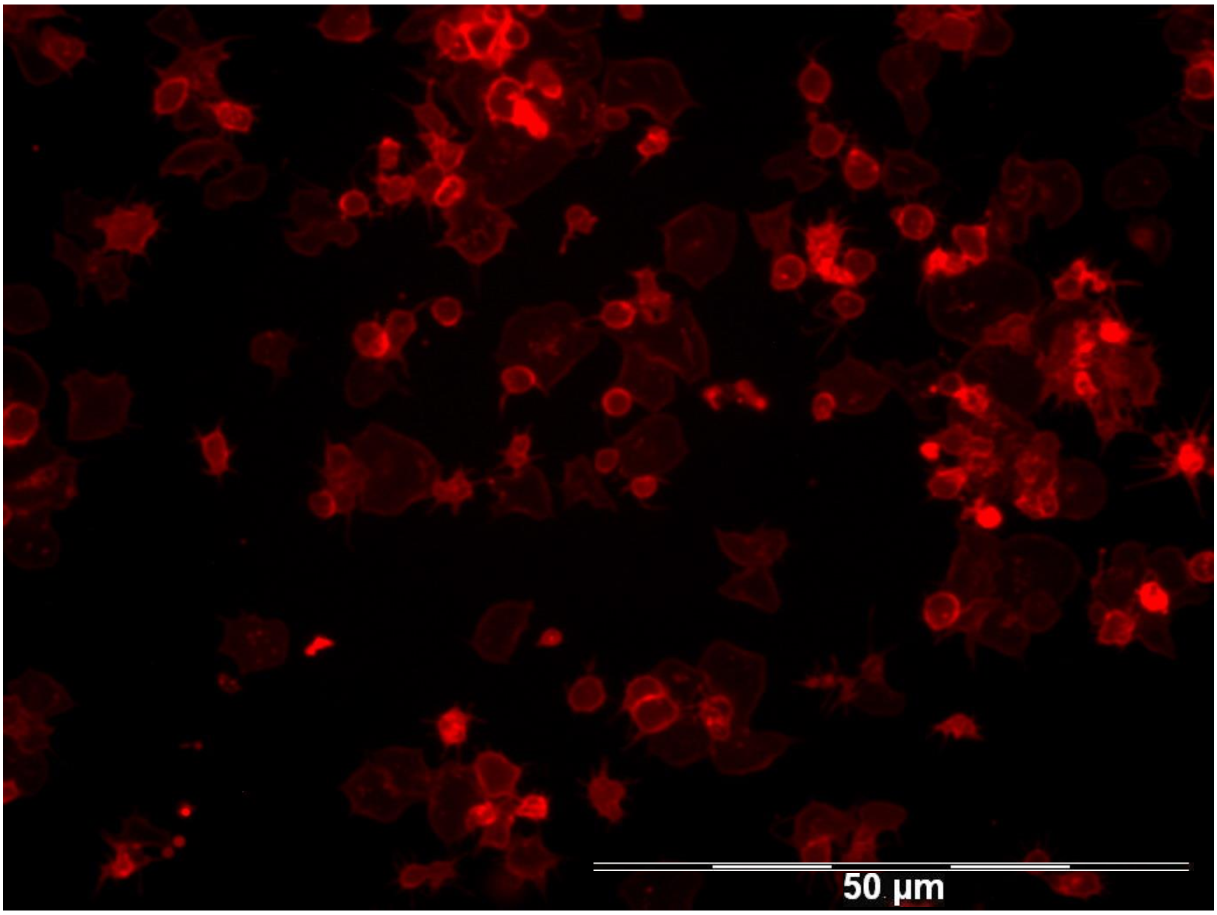

b

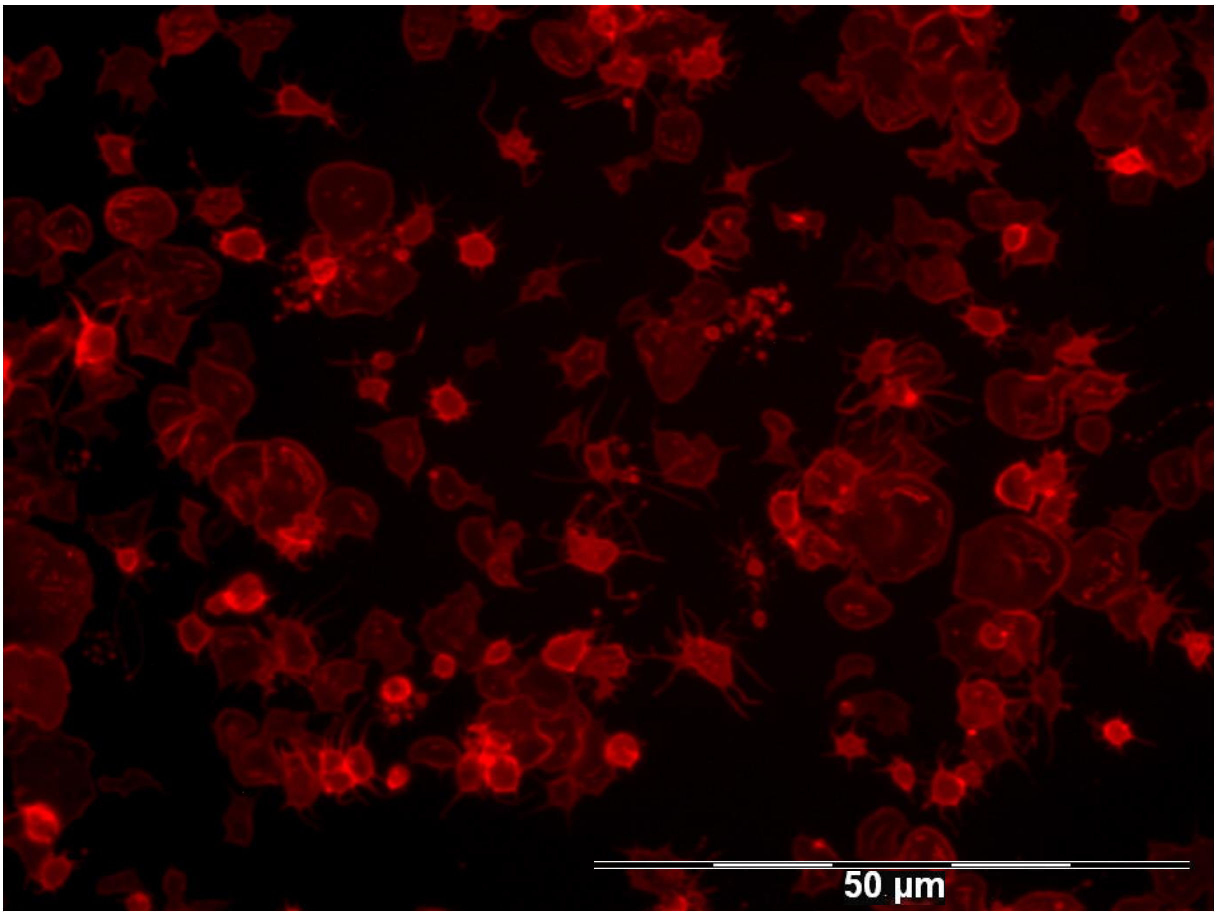

c

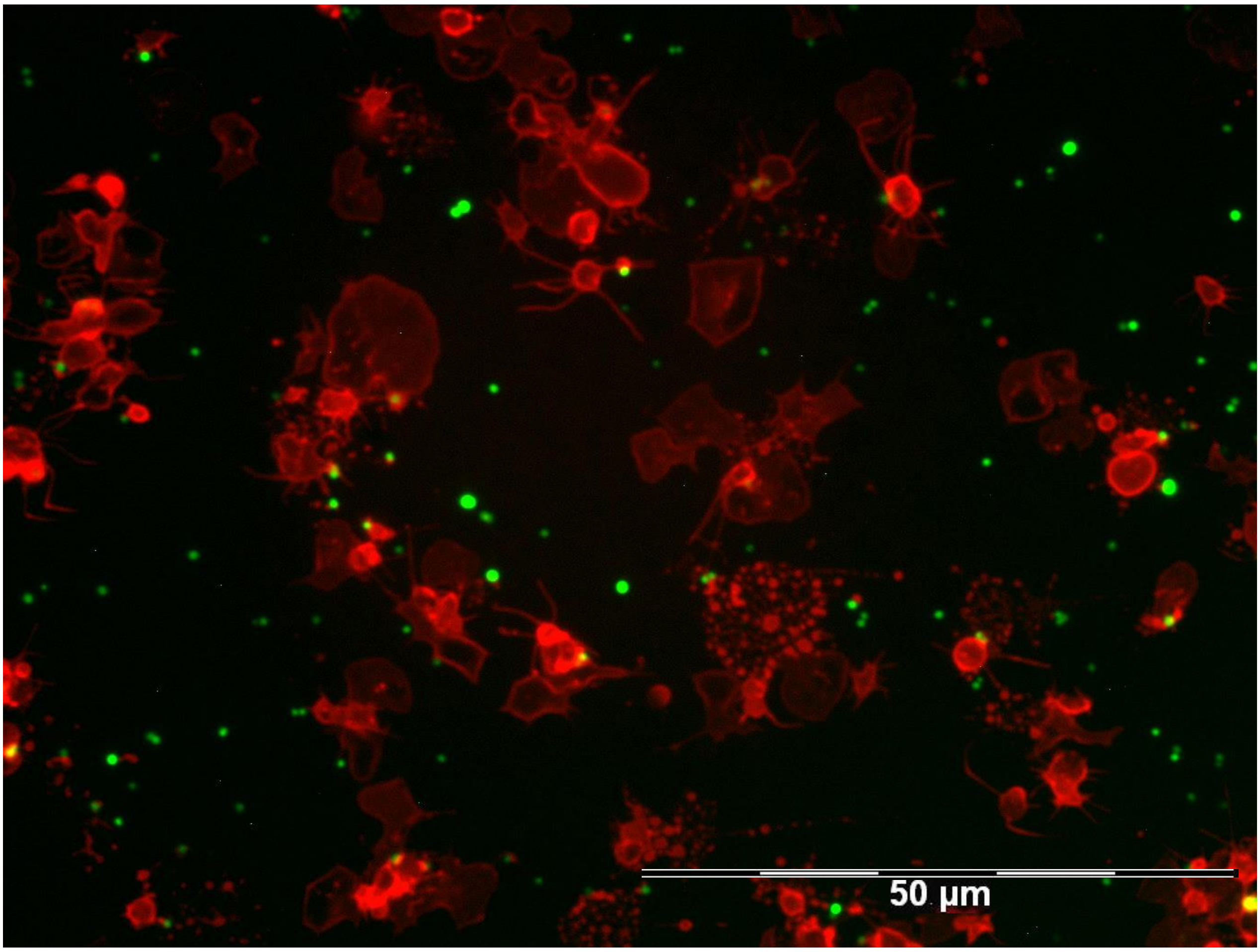

Supplemental Figure 1. ***S. aureus*** **SaB24 alters platelet morphology.** Platelets from healthy blood donors were stained with a Dylight 550 anti-CD41 antibody and analyzed by immunofluorescence microscopy after a 30-min exposure to **(a)** Tyrode's buffer (negative control), **(b)** TRAP (positive control), or **(c)** eGFP *S. aureus* clinical strain SaB24. Images are representative of 5 independent experiments.

Supplemental Figure 2

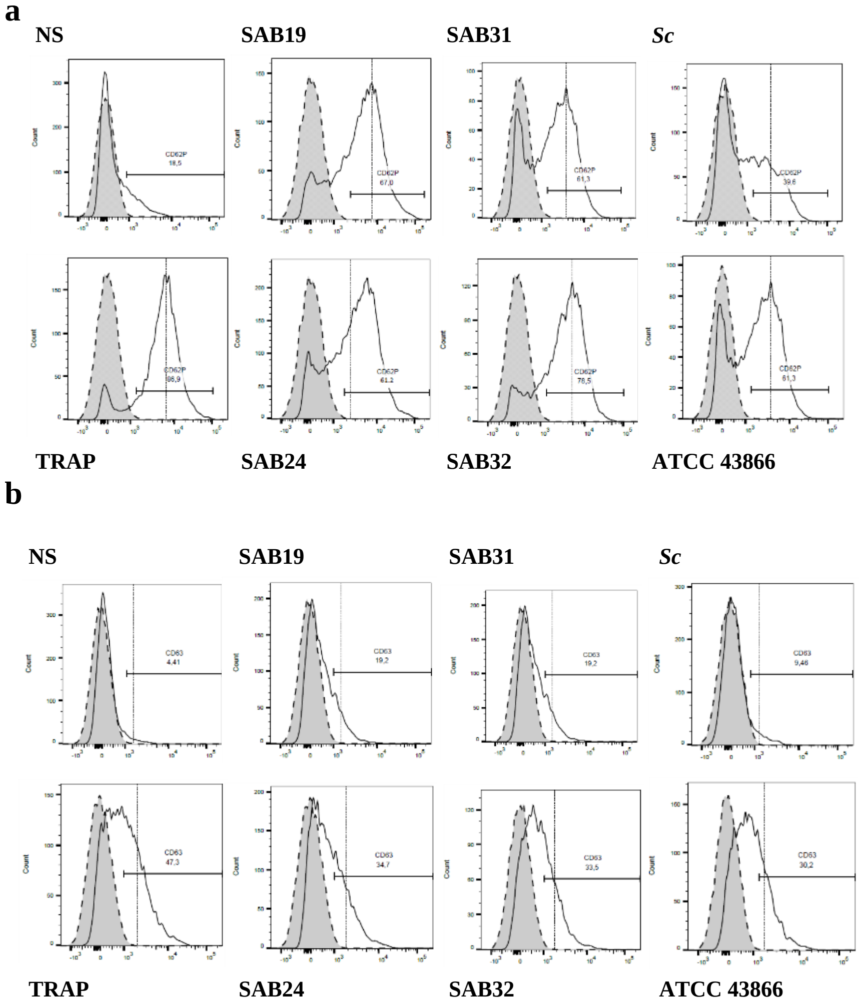

Supplemental Figure 2. Level of platelet activation after *Staphylococcus aureus* stimulation. Expression levels of the CD62P **(a)** and CD63 **(b)** activation markers on platelets were analyzed after stimulation for 30 min with staphylococcal strains [clinical strains (SaB), *S. condimenti* (Sc) or *S. aureus* ATCC 43866 (ATCC43866)], TRAP (as a positive control) or Tyrode's buffer (as a negative control, NS). Membrane expression of CD62P and CD63 after gating on CD41 was assessed by flow cytometry. One representative cytogram is presented for each condition with the isotype control (gray fill and dashed lines) and the marker CD62P or CD63 (white fill and solid lines).

Supplemental Figure 3

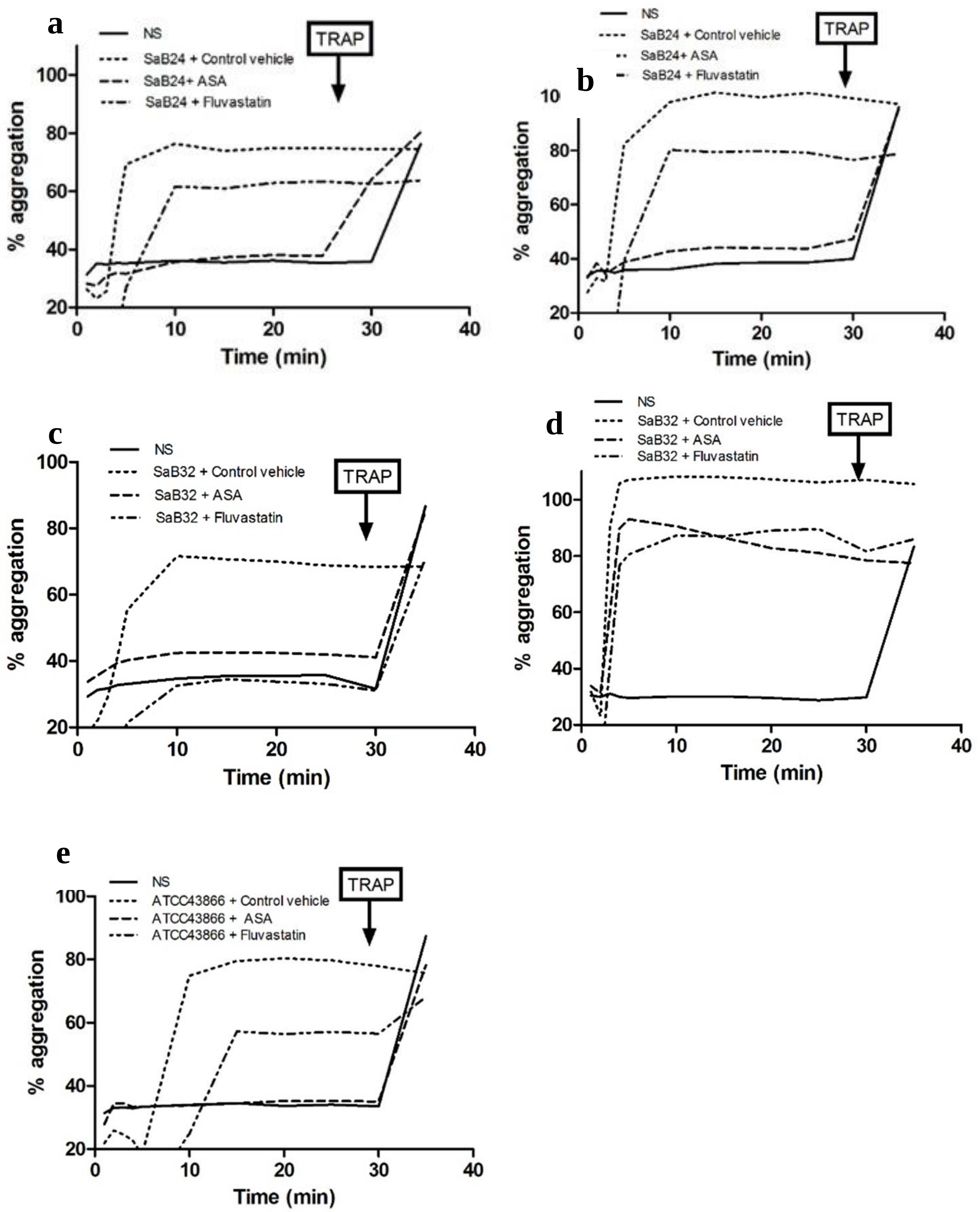

Supplemental Figure 3. Effect of ASA on *S. aureus*-induced platelet aggregation observed for 5/35 platelet samples.

Platelet aggregation was assessed using a thrombo-aggregometer following stimulation for 30 min with staphylococcal strains (**a**, SaB24; **b**, SaB24; **c**, SaB32; **d**, SaB32; **e**, ATCC43866), ASA,

fluvastatin or control vehicle (water). TRAP was added as a positive control at the end of the stimulation. The data from the 5/35 platelet samples that presented aggregation upon bacterial stimulation are shown. The aggregation baseline was approximately 20% due to the added volume.

Supplemental Figure 4

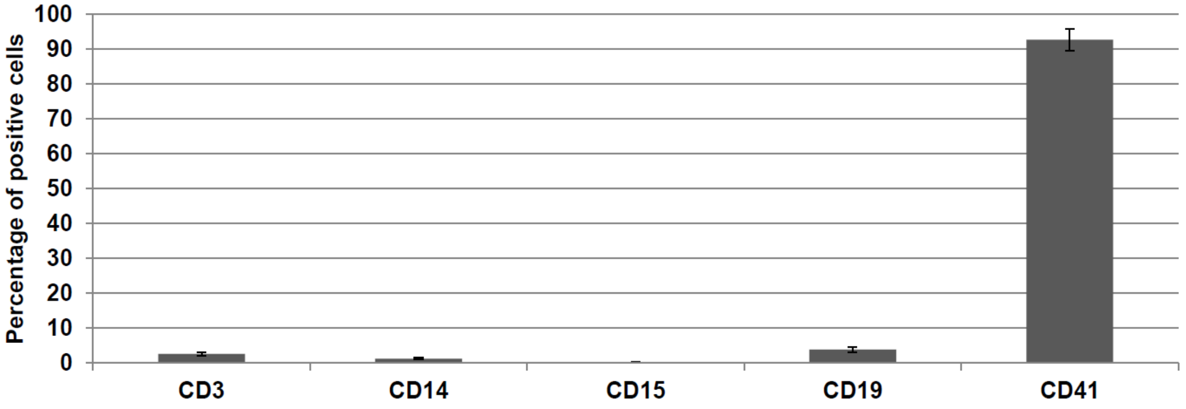

Supplemental Figure 4. Purity of the PRP preparation. Platelet purity was assessed using flow cytometry after staining cells with monoclonal antibodies against CD3 (specific marker for T cells), CD14 (specific marker for monocytes), CD15 (specific marker for neutrophils), CD19 (specific marker for B cells) or CD41 (specific marker for platelets). The data (mean ± SD; n = 5 experiments) are expressed as the percentage of positive cells.

Supplemental Figure 5

a

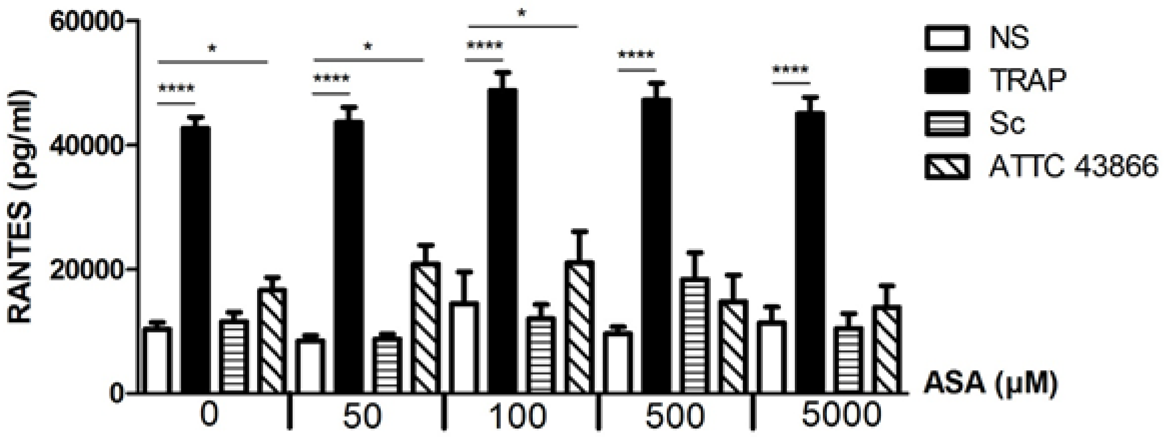

b

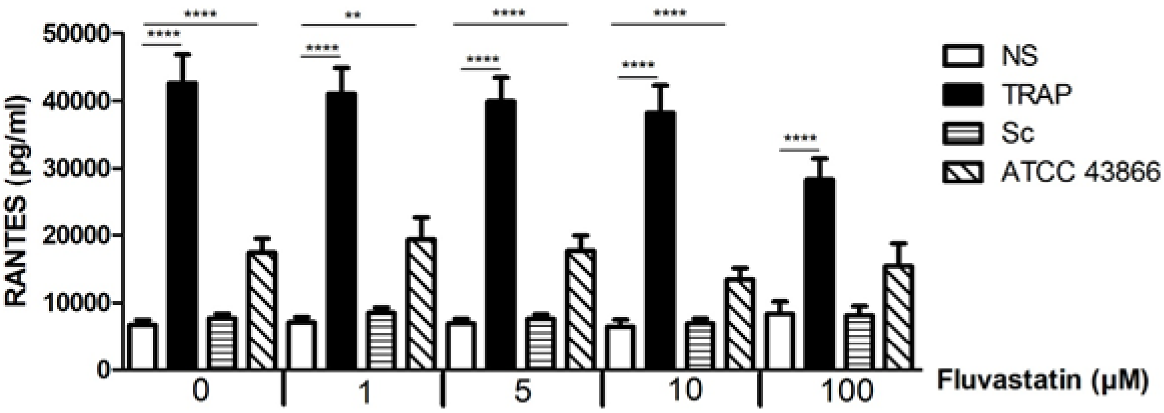

Supplemental Figure 5. Determination of the optimal concentrations of ASA and fluvastatin. RANTES release by platelets following a 30-min exposure to *S. condimenti* or *S. aureus* ATCC43866 strains (MOI of 0.1) in the presence of increasing concentrations of ASA (**a**, ranging from 0 to 5000 µM) or fluvastatin (**b**, ranging from 0 to 100 µM). *p<0.05; **p<0.01; ****p<0.0001 (two-way ANOVA with repeated measures and the Bonferroni post hoc test; stimulated *vs.* unstimulated, NS).

Supplemental Table 1

	SAB19	SAB24	SAB31	SAB32	ATCC43866
cap 1	-	-	-	-	-
cap 5	-	+	-	-	+
cap 8	+	-	+	+	-
icaA	+	+	+	+	+
icaC	+	+	+	+	+
icaD	+	+	+	+	+
bap	-	-	-	-	-
bbp	+	+	+	+	+
clfA	+	+	+	+	+
clfB (cons)	+	+	+	+	+
cna	+	+	-	-	-
ebh (cons)	+	+	+	+	+
ebpS	+	+	+	+	+
eno	+	+	+	+	+
FIBTOT	+	+	+	+	+
fnbA	+	+	+	+	+
fnbB	+	+	+	+	+
map	+	+	+	+	+
sasG	-	-	+	-	+
sdrC	+	+	+	+	+
sdrD	+	+	+	+	+
vwb	+	+	+	+	+
SSL-1total	+	+	+	+	+
SSL-2total	+	+	+	+	+
SSL-3total	+	-	+	+	+
SSL-4total	+	-	+	+	+
SSL-5total	+	+	+	+	+
SSL-6total	+	-	+	-	+
SSL-7total	+	+	+	+	+
SSL-8total	+	-	+	+	+
SSL-9total	+	+	+	+	+
SSL10total	+	+	+	+	+
SSL11total	+	-	+	-	+
SETB3total	+	+	+	+	+
SETB2tot	+	+	-	+	+
setB1	+	+	+	+	+
setC	+	+	+	+	+
Tst1 (consens)	-	-	-	-	-

	SAB19	SAB24	SAB31	SAB32	ATCC43866
entA	-	-	-	-	+
entA (N3+5) / entP	-	-	-	-	-
entB	-	-	-	-	-
entC	-	-	-	-	-
entD	-	-	-	-	-
entE	-	-	-	-	-
entG	-	-	-	-	-
entH	-	-	-	-	-
entI	-	-	-	-	nd
entJ	-	-	-	-	-
entK	-	-	-	-	-
entL	-	-	-	-	-
entM	-	-	-	-	-
entNTOT	-	-	-	-	-
entN (cons)	-	-	-	-	-
entN (other than RF122)	-	-	-	-	-
entO	-	-	-	-	-
entQ	-	-	-	-	-
entR	-	-	-	-	-
entU	-	-	-	-	-
entCM14 probe1	-	-	-	-	-
entCM+4 probe2	-	-	-	-	-
lukF	+	+	+	+	+
lukSTOT	+	+	+	+	+
lukS	+	+	+	+	+
lukS (ST22+ST45)	nd	+	+	+	-
hlgA	+	+	+	+	+
lukFndPV	-	-	-	-	-
lukSndPV	-	-	-	-	-
lukFndPV (P83)	-	-	-	-	-
lukM	-	-	-	-	-
lukD	+	-	+	+	+
lukE	+	-	nd	+	+
lukX	+	+	+	+	+
lukYTOT	+	-	+	+	+
lukY	+	nd	+	+	+
lukY (ST3-+ST45)	-	-	-	-	-
hl	+	+	+	+	+

	SAB19	SAB24	SAB31	SAB32	ATCC43866
hla	+	+	+	+	+
hlIIITOT	+	+	+	+	+
hlIII (cons)	+	+	+	+	+
hlIII (other than RF+22)	+	+	+	+	+
hlbTOT	+	+	-	+	+
hlbndprobe 1	+	nd	-	+	+
hlbndprobe 2	+	+	-	+	+
hlbndprobe 3	+	-	-	+	nd
unndtruncated hlb	-	-	-	-	-
sak	+	-	-	+	+
chp	-	+	+	-	-
scn	+	+	+	+	+
etA	-	-	-	-	-
etB	-	-	-	-	-
etD	-	-	-	-	-
edinA	-	-	-	-	-
edinB	-	-	-	-	-
edinC	-	-	-	-	-
arcAndSCC	-	-	-	-	-
arcBndSCC	-	-	-	-	-
arcCndSCC	-	-	-	-	-
arcDndSCC	-	-	-	-	-
aurTOT	+	+	+	+	+
aur (cons)	+	+	+	+	+
aur (Other than MRSA252)	+	-	+	+	+
aur (MRSA252)	-	+	-	-	-
splA	+	-	+	+	+
splB	+	-	+	+	+
splE	+	-	+	+	+
sspA	+	+	+	+	+
sspB	+	+	+	+	+
sspPTOT	+	+	+	+	+
sspP (cons)	+	+	+	+	+
sspP (other than ST93)	+	+	+	+	+
AGR	+	+	+	+	+

Supplemental Table 1. Genotyping of clinical and reference *S. aureus* strains.

The *S. aureus* reference strain ATCC43866 strain and 4 *S. aureus* clinical strains isolated from bacteremia patients (SaB) 19, 24, 31, 32 were genotyped with the *S. aureus* Genotyping Kit 2.0 (Alere Technologies GmbH, Jena, Germany). Briefly, strains were grown on Colombia blood agar, and the DNA was extracted, amplified and assessed as recommended by the manufacturer.
